# Epigenetic and transcriptomic characterization reveals progression markers and essential pathways in clear cell renal cell carcinoma

**DOI:** 10.1038/s41467-023-37211-7

**Published:** 2023-03-27

**Authors:** Yige Wu, Nadezhda V. Terekhanova, Wagma Caravan, Nataly Naser Al Deen, Preet Lal, Siqi Chen, Chia-Kuei Mo, Song Cao, Yize Li, Alla Karpova, Ruiyang Liu, Yanyan Zhao, Andrew Shinkle, Ilya Strunilin, Cody Weimholt, Kazuhito Sato, Lijun Yao, Mamatha Serasanambati, Xiaolu Yang, Matthew Wyczalkowski, Houxiang Zhu, Daniel Cui Zhou, Reyka G. Jayasinghe, Daniel Mendez, Michael C. Wendl, David Clark, Chelsea Newton, Yijun Ruan, Melissa A. Reimers, Russell K. Pachynski, Chris Kinsinger, Scott Jewell, Daniel W. Chan, Hui Zhang, Aadel A. Chaudhuri, Milan G. Chheda, Benjamin D. Humphreys, Mehdi Mesri, Henry Rodriguez, James J. Hsieh, Li Ding, Feng Chen

**Affiliations:** 1 Oncology Division, Department of Medicine, Washington University in St. Louis, St. Louis, MO 63110 USA; 2 McDonnell Genome Institute, Washington University in St. Louis, St. Louis, MO 63108 USA; 3 Department of Pathology and Immunology, Washington University in St. Louis, St. Louis, MO 63110 USA; 4 Department of Genetics, Washington University in St. Louis, St. Louis, MO 63110 USA; 5 Department of Mechanical Engineering and Materials Science, Washington University in St. Louis, St. Louis, MO 63130 USA; 6 Department of Pathology, Johns Hopkins University, Baltimore, MD 21231 USA; 7 Van Andel Institutes, Grand Rapids, MI 49503 USA; 8 The Jackson Laboratory for Genomic Medicine, 10 Discovery Drive, Farmington, CT 06032 USA; 9 Department of Neurology, Washington University School of Medicine, St. Louis, MO 63110 USA; 10 Office of Cancer Clinical Proteomics Research, National Cancer Institute, Bethesda, MD 20892 USA; 11 Department of Radiation Oncology, Washington University School of Medicine, St. Louis, MO 63110 USA; 12 Siteman Cancer Center, Washington University in St. Louis, St. Louis, MO 63110 USA

**Keywords:** Cancer genomics, Tumour heterogeneity, Tumour biomarkers, Renal cell carcinoma

## Abstract

Identifying tumor-cell-specific markers and elucidating their epigenetic regulation and spatial heterogeneity provides mechanistic insights into cancer etiology. Here, we perform snRNA-seq and snATAC-seq in 34 and 28 human clear cell renal cell carcinoma (ccRCC) specimens, respectively, with matched bulk proteogenomics data. By identifying 20 tumor-specific markers through a multi-omics tiered approach, we reveal an association between higher ceruloplasmin (*CP*) expression and reduced survival. *CP* knockdown, combined with spatial transcriptomics, suggests a role for CP in regulating hyalinized stroma and tumor-stroma interactions in ccRCC. Intratumoral heterogeneity analysis portrays tumor cell-intrinsic inflammation and epithelial-mesenchymal transition (EMT) as two distinguishing features of tumor subpopulations. Finally, *BAP1* mutations are associated with widespread reduction of chromatin accessibility, while *PBRM*1 mutations generally increase accessibility, with the former affecting five times more accessible peaks than the latter. These integrated analyses reveal the cellular architecture of ccRCC, providing insights into key markers and pathways in ccRCC tumorigenesis.

## Abstract (web summary)

Tumour heterogeneity in clear cell renal cell carcinoma (ccRCC) remains to be investigated. Here, the integration of spatial omics, transcriptional and chromatin accessibility profiling at the single-nucleus level and bulk proteogenomics data reveal markers and pathways important for ccRCC.

## Introduction

Renal cell carcinoma encompasses a variety of subtypes, among which clear cell renal cell carcinoma (ccRCC) is the most common form, comprising roughly 70% of cases^1^. Reliable tumor-cell markers are required for diagnosis and prognosis. Only a few immunohistochemical markers, such as carbonic anhydrase IX (CA9), vimentin (VIM), and CD10 have been used for the diagnosis of ccRCC^2,3^. So far, bulk gene expression profiling has helped discover biomarkers for renal tumor diagnosis^3^. As CAR T-cell therapy for ccRCC is emerging^4,5^ (NCT04696731, NCT04969354), there is a need for markers with specific expression in tumor cells^6^. Single-cell (sc) or single-nucleus (sn) RNA-seq helps overcome the issue of the averaged signal by bulk profiling methods, allowing for systematic evaluation of tumor markers within and across samples. Furthermore, spatial transcriptomics enables the characterization of the spatial heterogeneity of the transcriptome in conjunction with histopathological features, which was inaccessible by the bulk profiling methods.

ccRCC is considered a metabolic disease, accompanied by reprogramming of glucose and fatty acid metabolism^7–11^. Studies using genomic^12^, proteomic^7,8,13^, and metabolomic^14,15^ profiling have uncovered metabolic shifts in aggressive ccRCCs that involve the tricarboxylic acid cycle (TCA), pentose phosphate, and phosphoinositide 3-kinase pathways. However, our understanding of how some of the critical metabolic enzymes are up/down-regulated, specifically through transcriptional regulation in ccRCC, remains incomplete. Moreover, it is unclear whether tumor subpopulations are metabolically distinct and in which pathways.

*VHL* loss, the most frequent molecular alteration of ccRCC^12^, has been implicated to cause pervasive enhancer malfunction^16^. In addition, 80% of ccRCC tumors also carry non-synonymous mutations in epigenetic regulators and chromatin remodeling genes^17^. *BAP1* (BRCA1-associated protein 1) and *PBRM1* (Polybromo 1) are two of the most recurrently mutated genes in ccRCC after *VHL*. Mutations in them tend to be mutually exclusive^18–21^, although double mutants have been observed^12^. BAP1 is a deubiquitinase enzyme and a tumor suppressor protein. It removes Polycomb-mediated H2AK119ub1, an epigenetic mark essential for maintaining transcriptional repression^22^. BAP1 loss is associated with the accumulation of H2AK119ub1 and global chromatin condensation^23,24^. On the other hand, *PBRM1* encodes the tumor suppressor BAF180, which is a subunit of the nucleosome remodeling complex PBAF. Work by Kakarougkas et al. showed that the BAF180 is essential for the repair of DNA double-strand breaks by inhibiting transcription globally^25^. Previous studies have suggested that *BAP1* and *PBRM1* mutations are associated with non-overlapping gene expression signatures, differential mTORC1 activation, and different patient outcomes^18,19^, the former associated with worse survival than the latter. However, the consequences of *BAP1* and *PBRM1* mutations on overall tumor cell chromatin accessibility and associated transcriptome changes in ccRCC are largely unknown.

Aside from these regulation and remodeling dynamics, ccRCC is known for its substantial genetic heterogeneity, parallel evolution of subclones^26^, and abundant genetic alterations, as revealed by bulk sequencing-based studies^27,28^. However, whether there are correspondingly high levels of heterogeneity in the transcriptome and chromatin accessibilities in ccRCC cells remains largely unknown, as do the most distinguishing features of intrapatient tumor subpopulations. So far, scRNA-seq studies of relatively small numbers of ccRCC samples have been reported, shedding light on the molecular attributes of tumor cells of origin^29,30^, tumor microenvironments, tumor markers^29,31^, and therapeutic targets^32^. However, scRNA-seq has been limited by the availability of fresh tissue, especially since clinical samples are normally cryo-preserved^33^. Single nucleus RNA-seq (snRNA-seq) can analyze frozen specimens and avoids the cell dissociation process that promotes stress-related alterations^34^.

In this work, we integrate snRNA-seq, snATAC-seq, bulk omics (including proteomics), and spatial transcriptomics (ST) to investigate the epigenetic and transcriptomic landscape of ccRCC. We identify key markers specifically altered both epigenetically and transcriptionally in tumor cells, particularly *CP*. Using spatial transcriptomics and *CP*-knockdown cell lines, we find that spatial distribution of *CP* gene expression is associated with *COL4A1* expression and hyalinized stroma. We discover transcription factors regulating *CP* and glycolytic genes, which are differentially accessible and expressed between tumor cells and normal proximal tubule cells. We also map tumor subpopulations and demonstrate the differential activity and epigenetic regulation of a few key pathways. Finally, we dissect chromatin accessibility changes associated with *BAP1* and *PBRM1* mutations, further illustrating the multi-level interplay between mutational, global, and specific epigenetic alterations, and transcriptomic changes in ccRCC.

## Results

### Overview of clinical features and datasets

**Fig. 1:**
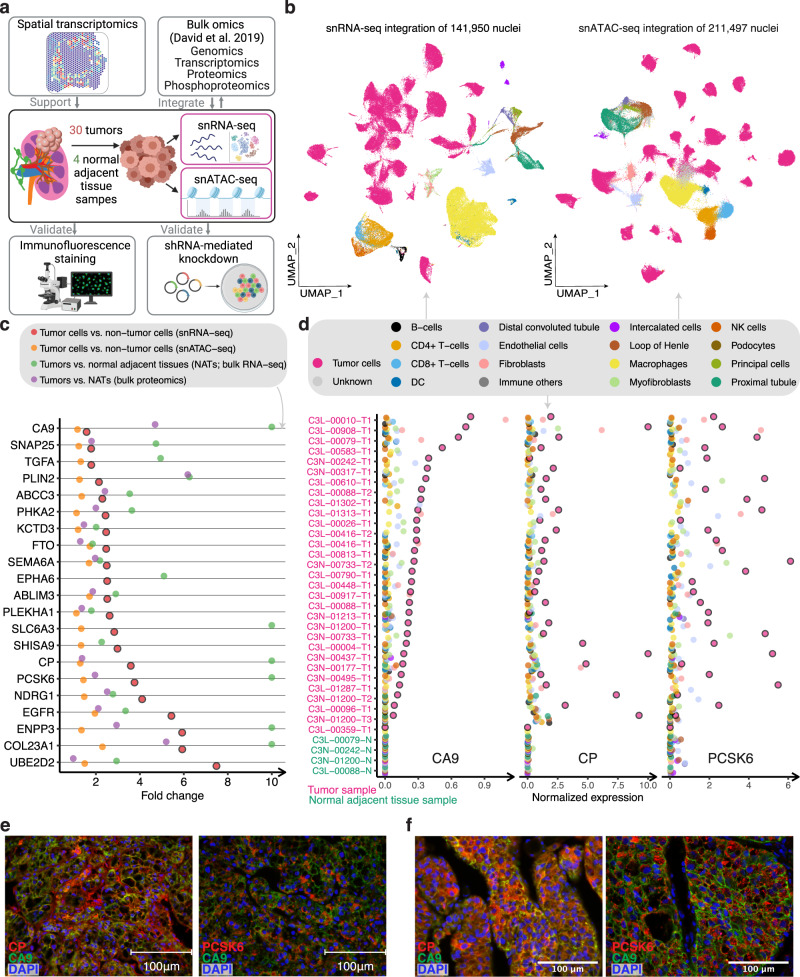
snRNA-seq analysis identifies tumor-cell-specific markers. **a** Schematic for integrating snRNA-seq, snATAC-seq, bulk omics, and spatial transcriptomics data, and validating the omics findings with immunofluorescence staining and shRNA-mediated knockdown experiments (image is created with BioRender.com. **b** UMAP visualization of 141,950 nuclei and 211,497 nuclei profiled by snRNA-seq and snATAC-seq, respectively, colored by major cell groups. The cell group named “immune others” includes basophils, mast cells, lymphoid lineage immune cells with proliferating signature, and immune cells with ambiguous myeloid/lymphoid identity. **c** Dot plot showing the fold changes of expression of tumor-cell markers in ccRCC (capped at 10). Red dots denote the gene expression fold changes between tumor cells vs. non-tumor cells using snRNA-seq data. Orange dots denote the gene accessibility fold changes between tumor cells vs. non-tumor cells using snATAC-seq data. Green dots denote the gene expression fold changes between bulk tumor and normal adjacent tissue (NAT) samples using bulk RNA-seq data. Purple dots denote the bulk protein level changes (spectrum intensity) between tumors vs. NAT samples. **d** Dot plot showing the expression levels of *CA9*, *CP*, and *PCSK6* in each cell type and each sample (non-log space). Expression levels for tumor cells are highlighted by black outlined circles. Immunofluorescence (IF) staining of a ccRCC **e** patient tumor sample (ID: 293) and **f** patient-derived xenograft tumor (ID: RESL5). Left panel, markers CP (red), CA9 (green), and DAPI (nucleus, blue). Right panel, markers PCSK6 (red), CA9 (green), and DAPI (nucleus, blue). Scale bars in the (**e**, **f**) are 100 μm. Three independent experiments were performed with similar results. Scale Source data are provided as a Source data file.

We performed snRNA-seq on 34 samples (25 patients) and matched snATAC-seq on 28 of these samples (24 patients) from the Clinical Proteomic Tumor Analysis Consortium (CPTAC) ccRCC collection^13^ (Supplementary Data 1). Source materials were procured from the same pools of pulverized powder that previously produced WES, bulk RNA-seq, and proteomics data for these samples (Fig. 1a, Supplementary Fig. 1a). Common sourcing ensures the highest level of comparability among datasets from different platforms and enables tight integration of diverse omics datasets. We also performed spatial transcriptomics (ST) on 2 patient tumor samples collected in-house using the FFPE Visium ST platform (10x Genomics).

Regarding snRNA-seq data, we obtained 141,950 nuclei from 34 samples, comprising 30 primary tumor samples and 4 normal adjacent tissue (NAT) samples (Fig. 1b). These samples displayed distinct tumor, immune, and stromal populations based on canonical markers curated from the literature (Supplementary Data 2). Confirmation of known mutational and transcriptional alterations in ccRCC cells (Supplementary Fig. 1b–d) validates these single-nuclei methods as powerful tools to study cancer cell behavior at high resolution. The snRNA-seq data indicated tumor cell content averaged 71% per sample, which correlated strongly with the bulk mRNA data estimate (Pearson’s R = 0.58, *P* < 0.001, Supplementary Fig. 1e). We also generated snATAC-seq data for 211,497 nuclei from 24 of these same tumors and 4 NATs (Fig. 1b). We detected peaks of accessible chromatin in snATAC-seq data across all samples, ranging from 86 K to 220 K instances per sample. As expected^35^, the majority of peaks appeared in intronic and intergenic regions, while an average of 24 K peaks were located in gene promoter regions (Supplementary Fig. 1f). snRNA and snATAC paired samples yielded comparable cell type content estimates (Pearson’s R = 0.77, *P* < 0.0001, Supplementary Fig. 1e).

### Single-cell-based ccRCC tumor marker discovery and epigenetic regulation of tumor markers

Although bulk sequencing studies have reported markers altered between ccRCC and adjacent normal tissue that presumably reflect changes in tumor cells^12^, those studies were limited by confounding effects from non-tumor cells or had limited discovery power for subpopulations of tumor cells expressing unique markers. We sought to discover tumor-cell markers that are specific to ccRCC tumor cells and may have prognostic/diagnostic values or potential therapeutic targets. Here, we performed a 4-stage process to identify bona fide markers for ccRCC by leveraging snRNA-seq, snATAC-seq, bulk RNA-seq, and proteomics data (Supplementary Fig. 2a). We identified 324 ccRCC tumor-cell-specific markers from the snRNA-seq analysis (Supplementary Data 3), among which we prioritized 20 markers (Fig. 1c) that are significantly higher in tumor cells than proximal tubule cells (considered the ccRCC cell of origin; “Methods”). 19 of these showed higher chromatin accessibility (gene activity, fold change (FC) > 1) in tumor cells using snATAC-seq data, suggesting higher chromatin accessibility may contribute to their higher expression in tumor cells. Of these, 17 were further supported by the bulk RNA-seq and proteomics data (*n* = 103), as evidenced by comparing the tumors to the normal adjacent tissues of a larger cohort (Fig. 1c).

Two interesting examples are ceruloplasmin (*CP)* and Proprotein Convertase Subtilisin/Kexin Type 6 (*PCSK6*). *CP* is a reported tumor marker for ccRCC^36–38^ but *PCSK6* is not. Their tumor cell-specific expressions using snRNA-seq (tumor cells vs. non-tumor cells fold change = 3.6 and 3.7, respectively) are illustrated in Fig. 1d. Co-localization of CP and CA9 proteins using IF staining validated tumor cell-specific *CP* expression (Fig. 1e, f). We also validated the tumor cell expression of PCSK6 (Fig. 1f). High tumor-cell expression levels of CP and PCSK6 (from snRNA-seq) are associated with higher tumor grades (FDR < 1e−10; G3 and G4 vs. G1 and G2). A significant difference in CP level was also observed between high-grade and low-grade tumors using bulk RNA-seq data (FDR < 0.01), but not for PCSK6 (Supplementary Fig. 2b–d). High *CP* expression is also associated with shorter overall survival in this study cohort and the larger ccRCC CPTAC cohort with bulk RNA-seq data (Supplementary Fig. 2e). However, PCSK6 expression is not significantly associated with patient survival using snRNA-seq data (Supplementary Fig. 2f). These results suggest that *PCSK6* and *CP* are promising tumor cell markers and *CP*, in particular, could be of potential diagnostic and prognostic values in ccRCC.

### CP in mediating tumor extracellular matrix and tumor-stroma interaction in ccRCC

**Fig. 2:**
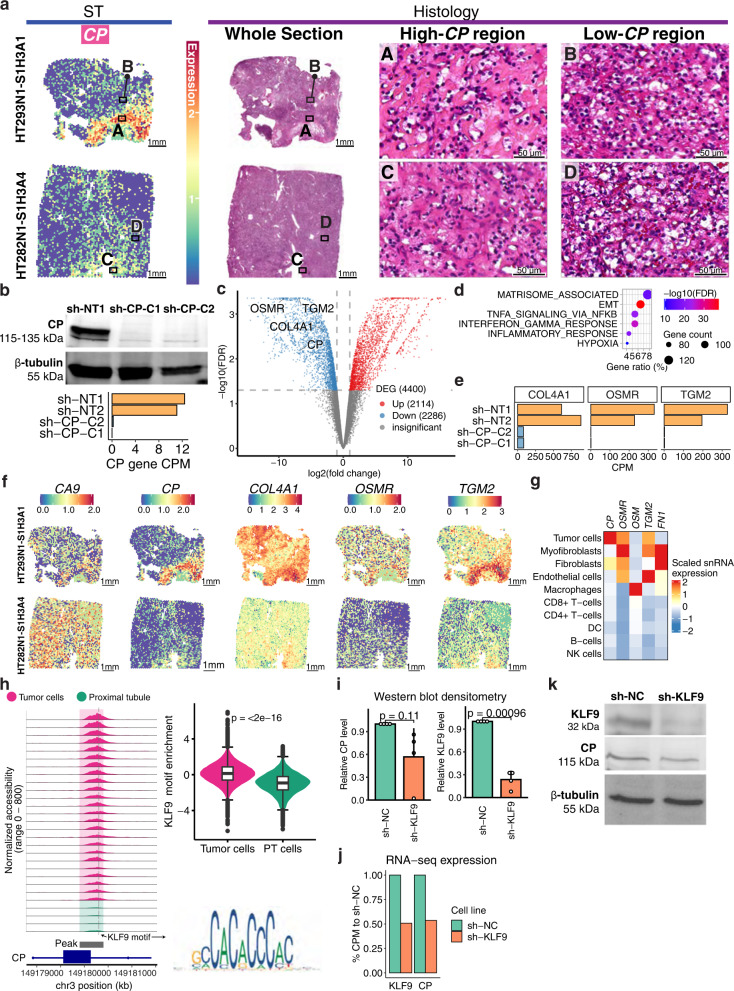
Ceruloplasmin (CP) spatial expression pattern and CP knockdown effect on the transcriptome in ccRCC cells. **a** Spatial transcriptomics and H&E histology of two ccRCC specimens. Regions showing high-*CP* (regions A and C) and low-*CP* (regions B and D) expression were indicated. Scale bars are 1 mm. **b** Top: Western blot of CP and β-tubulin on proteins from Caki-1 cells expressing *CP* shRNA (sh-CP-C1, sh-CP-C2) and non-transduced Caki-1 cells (sh-NT1). Three independent experiments were performed that showed similar results. Bottom: Bar plot showing normalized bulk gene expression of *CP*. **c** Volcano plot showing differentially expressed genes between Caki-1 cells with *CP* knockdown (sh-CP-C1 and sh-CP-C2) vs. cells without *CP* knockdown (sh-NT1 and sh-NT2). Statistical evaluation was performed using two-sided edgeR analysis with glmQLFTest followed by multiple testing correction (Benjamini–Hochberg). **d** Bubble plot showing the pathways over-represented in genes downregulated (top) in the *CP*-knockdown lines (sh-CP-C1 and sh-CP-C2) vs. controls (sh-NT1 and sh-NT2). The p.adjust represents Benjamini–Hochberg adjusted P-value from one-sided Fisher’s exact test. **e** Bar plot showing normalized bulk gene expression of *COL4A1*, *OSMR*, and *TGM2* in sh-CP-C1, sh-CP-C2, sh-NT1, and sh-NT2. **f** Spatial transcriptomics for *CA9*, *CP*, *COL4A1*, *OSMR*, and *TGM2*. **g** Heatmap showing scaled snRNA-seq expression of *CP*, *OSMR*, *OSM*, *TGM2*, and *FN1* across cell types. **h** Left: Genomic region near *CP* gene promoter (in ccRCC cells). The plots show the normalized accessibility by snATAC-seq around these regions in proximal tubule cells (green; *n* = 123,35) from NAT samples and tumor cells (pink; *n* = 119,191) from tumor samples. Top right: Violin plot showing the distribution of KLF9 motif enrichment scores in tumor cells (*n* = 119,191) and PT cells (*n* = 123,35). The box bounds the interquartile range divided by the median, with the whiskers extending to a maximum of 1.5 times the interquartile range beyond the box. Outliers are shown as dots. Student’s T-test; *P* value is two-sided. Bottom right: position weight matrix for KLF9 motif. **i** Bar plot showing western blot densitometry of KLF9 and CP proteins in RCC4 cells expressing KLF9 shRNA (sh-KLF9) and RCC4 cells expressing scrambled control (sh-NC). The error bar represents the standard deviation of the mean from four independent experiments performed. Wilcoxon rank-sum tests; P values are two-sided. **j** Bar plot showing % of normalized gene expression of *KLF9* and *CP* compared to the control. **k** Western blot showing KLF9, CP, and β-tubulin protein levels in sh-NC and sh-KLF9. Four independent experiments were repeated and showed similar results (as shown in **i**). Source data are provided as a Source data file.

To understand the spatial distribution of tumor markers, we generated spatial transcriptomics (ST) data to validate selected tumor markers in two ccRCC tumor tissue samples. *CP* expression exhibited a spatially-dependent enrichment pattern in both samples (Fig. 2a). Tumor 293 displayed enriched *CP* expression in an area showing a relative sparsity of tumor cells embedded in an abundant background of hyalinized stroma (location A) compared to the rest of the tumor (location B). Tumor 282 also showed higher *CP* expression in an area showing a higher hyalinization-to-cell ratio (location C) than in the rest of the cancer (location D), indicating partial regressive changes. These observations suggest *CP* may have a role in the hyalinization of the tumor microenvironment and possibly mediating tumor-stroma interactions.

Despite being a ccRCC tumor marker, we know little about the molecular pathways and genes regulated by *CP* in ccRCC. Therefore, we generated two derivatives of Caki-1 cell lines with reduced *CP* expression using shRNA-mediated gene suppression. Caki-1 cells expressing CP shRNA constructs (sh-CP-C1 and sh-CP-C2) had reduced *CP* transcripts (Fig. 2b bottom panel) and protein abundance (Fig. 2b top panel) compared to the non-transduced control (sh-NT1). We identified 4400 differentially expressed genes (DEGs; FDR < 0.05, |log_2_FC| > 1) between Caki-1 cells with or without *CP* knockdown (Fig. 2c, Supplementary Data 3) using bulk RNA-seq. Gene sets most enriched in downregulated genes include matrisome-associated, epithelial–mesenchymal transition, TNF-α signaling via NF-κB, interferon-gamma response, and inflammatory response (Fig. 2d).

Down-regulation of matrisome-associated and inflammatory response genes associated with *CP* knockdown supported our hypothesis that *CP* may have a role in mediating tumor-stroma interactions. Here, collagen type IV alpha1 chain (COL4A1) showed decreased expression in *CP*-knockdown lines (Fig. 2e), which also showed enrichment in *CP*-high regions in the ST data (Fig. 2f). Collagen IV is the primary extracellular matrix material contributing to hyalinized stroma^39^. These observations suggest that high *CP* expression in ccRCC tumor cells mediates the secretion of collagen IV, contributing to a more hyalinized stroma. In addition, the oncostatin M receptor (*OSMR*) is downregulated with *CP* knockdown (Fig. 2e). *OSMR* showed relatively high expression in tumor cells compared to other cell types (Fig. 2g). Our snRNA-seq data also showed that macrophages express *OSMR*’s major ligand oncostatin M (*OSM*) (Fig. 2g), suggesting that ccRCC tumor cells may interact with macrophages through OSM signaling transduction. *OSMR* is highly expressed in *CP*-high regions in the ST data (Fig. 2f), while *OSM* expression is less spatially enriched (Supplementary Fig. 2g). These results suggest that tumor marker CP may regulate OSM-OSMR signaling between tumor cells and macrophages. Similarly, we also observed an association between CP and TGM2-FN1 signaling between tumor cells and myofibroblasts (Fig. 2e–g). TGM2 is downstream of OSMR signaling^40^. As TGM2-FN1 signaling and COL4A1 are also associated with cell migration and invasion in cancer^40,41^, CP may also have a role in influencing the migration of ccRCC cells. Our results extend beyond the current knowledge about CP being a ccRCC tumor marker and shed light on the molecular functions of CP in promoting ccRCC pathogenesis.

Previous studies have demonstrated that HIF1A and PAX8 activate the transcription of the *CP* gene^42,43^. Here, our data suggested KLF9 may also be a transcription factor (TF) regulating *CP* transcription: we found a KLF9 binding motif in a *CP* open promoter region (Fig. 2h), which is more accessible in ccRCC cells compared to their normal counterparts (proximal tubule cells). KLF9 motif binding accessibility is significantly enriched in ccRCC cells based on snATAC-seq data (Fig. 2h), suggesting that KLF9 is an active transcription factor in ccRCC cells. We generated a derivative of the RCC4 cell line with shRNA-mediated knockdown of *KLF9* expression (sh-KLF9; Fig. 2i–k), reducing the KLF9 protein by 70% of that in RCC4 cells expressing a control scrambled shRNA (sh-NC; *P* = 0.0025). We observed a concomitant decrease in *CP* transcript and protein in the *KLF9*-knockdown line compared to the scrambled control, although the p-value is not significant (*P* = 0.11). These data nominate KLF9 as a transcription regulator of *CP* and warrant its further experimental validation.

### Transcription factors mediating glycolytic genes in ccRCC cells

To ascertain the closest normal epithelial counterparts for ccRCC cells, we examined transcription factor (TF) motif enrichment in different epithelial cell types based on TF motif binding accessibility (“Methods”). This differs from previous strategies that use gene expression and mutational analyses to identify the cell of origin for ccRCC cells. We found that ccRCC tumor cells had the strongest correlations with proximal tubule cells in TF binding accessibility (Supplementary Fig. 3a, left panel). This was supported by correlation analysis using snRNA-seq data (Supplementary Fig. 3a, right panel). Our data thus support the hypothesis that ccRCC derives from PT cells^29,44^ by using similarity in epigenetic regulation between tumor cells and PT cells, which adds to other approaches using gene expression and mutational analyses.

**Fig. 3:**
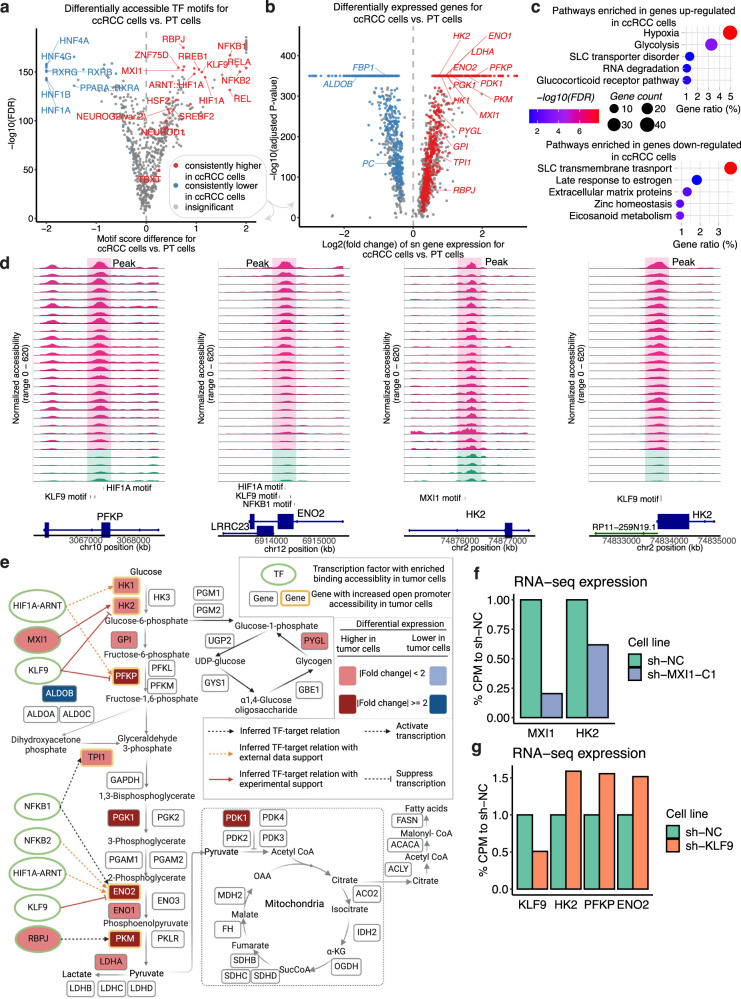
The glycolysis pathway displays significant changes in ccRCC tumor cells compared to the proximal tubule cells. **a** Volcano plot showing differentially enriched TF motifs between ccRCC (tumor) cells (*n* = 118,409) from 30 tumor samples and the combined proximal tubule (PT) cells from the four NATs (*n* = 9676). The X-axis shows the motif score difference, while the Y-axis shows the −log_10_(adjusted *P*-value). Statistical evaluation was performed using a two-sided Wilcoxon rank-sum test, applying Benjamini–Hochberg correction for the resulting *P*-values. Color denotes whether a motif is consistently higher or lower in tumor cells when tumor cells from individual tumor samples were compared to the PT cells or if it has insignificant or inconsistent fold changes (“Methods”). The motifs that have consistent higher/lower TF binding accessibilities in all comparisons of individual tumor vs. PT cells are highlighted. **b** Volcano plot showing differentially expressed genes between ccRCC cells (*n* = 88,536) and the combined PT cells from the NATs (*n* = 4269). The X-axis shows the log_2_(fold change) of the sn gene expression of the ccRCC cells compared to PT cells; the Y-axis shows the −log_10_(adjusted *P*-value). Statistical evaluation was performed using a two-sided Wilcoxon rank-sum test, applying Bonferroni correction for the resulting *P*-values. Color denotes whether a gene is consistently expressed higher or lower in tumor cells, or has insignificant or inconsistent fold changes (“Methods”). Genes for ccRCC-specific TFs and selected metabolic genes with significant fold changes are highlighted. **c** Bubble plot showing the pathways over-represented in genes upregulated (top) and downregulated (bottom) in ccRCC cells compared to the PT cells. The FDR represents Benjamini–Hochberg adjusted *P*-value from one-sided Fisher’s exact test. **d** Genomic regions near three upregulated genes in ccRCC cells compared to PT cells. The plots show the normalized accessibility by snATAC-seq around these regions in proximal tubule cells (green) from NAT samples and ccRCC cells (pink) from tumor samples. **e** When viewed in the context of important metabolic pathways in ccRCC, ccRCC cells displayed an overall upregulation of genes encoding glycolysis enzymes as well as other metabolic proteins (rounded rectangle) at sn gene expression level (red and blue filled colors represent significantly increased or decreased sn expression in ccRCC cells vs. proximal tubule cells). Among them, genes showing increased promoter accessibility are highlighted by the yellow border. Ellipses with green borders represent transcription factors with significantly enriched accessibility binding in ccRCC cells vs. PT cells. Lines connecting TFs and genes represent TF-target relations inferred by the presence of the TF motif in the more accessible promoter region of the genes using snATAC-seq. Black dotted lines denote inferred TF-target relation based on the snATAC-seq data in this study. Orange dotted lines denote those relations with literature support. Red solid lines denote those relations with experimental validation done in this study. Created with BioRender.com. **f** Bar plot showing the bulk RNA-seq expression of RCC4 cells with and without MXI1 knockdown. **g** Bar plot showing the bulk RNA-seq expression of RCC4 cells with or without KLF9 knockdown. Source data are provided as a Source data file.

We further identified 16 TF motifs that are most consistently enriched in tumor cells, including HIF1A/ARNT, NF-κB TFs (NFKB1, NFKB2, REL, RELA), RBPJ, MXI1, KLF9, ZNF75D, HSF2, NEUROD1, SREBF2, NEUROG2, RREB1, and TBXT (Fig. 3a). High HIF1A motif accessibility is consistent with the activation of HIF1A downstream transcriptional programs associated with *VHL* loss^45^. 14 of the 16 tumor-cell-specific TF motifs (except for SREBF2 and TBXT) were ccRCC-specific using the TCGA pan-cancer bulk ATAC-seq data^46^ (Supplementary Fig. 3b). We found that the expressions of *MXI1* and *RBPJ* were significantly upregulated in ccRCC cells using snRNA-seq data (Fig. 3b) and that the upregulation of RBPJ was further supported by bulk protein data (Supplementary Data 3). It appears that the activity of these TFs in ccRCC is not only enhanced by increased binding accessibility but also by increased TF abundance. Finally, HNF and RXR family TFs, which were more enriched in PT cells compared to ccRCC cells (Fig. 3a), were previously associated with ccRCC by bulk ATAC analysis^46^ (Supplementary Fig. 3c). These results highlight the utility of snATAC-seq in discerning motifs specific to tumor cells, as bulk ATAC analysis may confuse TFs specific to the normal PT cells with TFs specific to tumor cells.

To further investigate genes regulated by ccRCC-specific TFs, we identified 1161 overexpressed and 171 downregulated genes in ccRCC tumor cells in comparison to PT cells (Fig. 3b; Supplementary Data 3). The tumor-cell-overexpressing genes were enriched in glycolysis, hypoxia, solute carrier (SLC) transporter disorder, RNA degradation, and glucocorticoid receptor pathways (Fig. 3c). Because ccRCC is characteristic of the Warburg effect and is known to have glycogen and lipid accumulation^47^, we further investigated the transcriptional regulations for genes in glycolysis, TCA cycle, and glycogen and fatty acid synthesis pathways. A majority of the glycolysis enzymes were overexpressed in ccRCC cells, as was *PDK1*, which inhibits the conversion of pyruvate to acetyl-CoA in the mitochondria. It is worth noting that several gluconeogenesis enzymes, including fructose-1,6-bisphosphatase (*FBP1*) and pyruvate carboxylase (*PC*), were downregulated in ccRCC cells (Supplementary Data 3). These results support the view that glycolysis is activated in ccRCC cells^48,49^.

Previous studies have demonstrated that transcription factors, such as HIF-1, c-MYC, p53^50^, and SIX1^51^ play direct roles in regulating aerobic glycolysis. In our snATAC-seq data, we also observed motifs of HIF1A in the *PFKP, ENO2*, and *HK1* open promoter regions that are more accessible in tumor cells, consistent with the previous reports of HIF1A regulating these genes^52–55^ (Fig. 3d, Supplementary Data 3). In addition, we predicted that MXI1 might regulate the transcription for *HK2*, based on the presence of the MXI1 motif in an *HK2* open intronic region (Fig. 3d). We also predicted that KLF9 might regulate *PFKP*, *ENO2*, and *HK2* transcription, as we observed KLF9 motifs in the promoter regions of these genes. We generated an *MXI1*-knockdown cell line (Supplementary Fig. 3d, e) and performed RNA-seq. Here, the *MXI1*-knockdown lines showed lower *MXI1* and *HK2* expression than the scrambled control, suggesting *HK2* is regulated by MXI1. On the other hand, the *KLF9*-knockdown cell line showed an increase in *HK2*, *PFKP*, and *ENO2* expression compared to the control (Fig. 3g). Previous studies found that KLF9 could function as a transcription suppressor^56,57^. Thus KLF9 may be responsible for inhibiting the transcription of glycolytic genes (*HK2*, *PFKP*, and *ENO2*) in ccRCC. These results nominate candidate transcription factors regulating glycolytic genes in ccRCC and additional experimental validations are warranted.

### Transcriptome-based tumor-cell subclusters may represent genomically distinct subclones

**Fig. 4:**
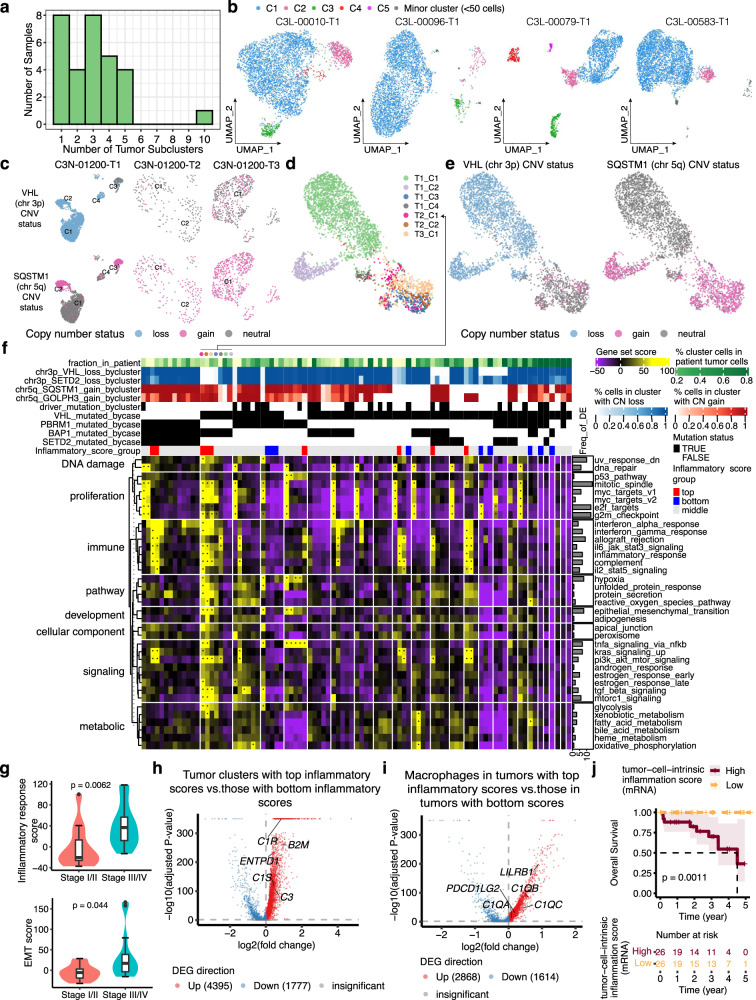
Intratumor signaling heterogeneity revealed by single-cell tumor subclustering. **a** Bar plot showing the number of tumor-cell clusters per sample. **b** UMAP illustration of the tumor-cell clusters for four tumor samples, colored by the cluster name. **c** UMAP showing tumor clusters of three tumor samples from the same patient, colored by the copy number status of *VHL* and *SQSTM1*. **d** UMAP showing merged data for tumor cells from the above three tumor samples, colored by the original tumor cluster name. **e** UMAP showing the copy number status of VHL and SQSTM for the merged data shown in (**d**). **f** Heatmap showing the gene set scores for 90 tumor subclusters (columns). Tumor subclusters are grouped by patient and separated by white lines. **g** Violin plot showing maximum inflammatory response score (top) and EMT score (bottom) per tumor sample, grouped by tumor stage (stage I/II: *n* = 12; stage III/IV: *n* = 22). The box bounds the interquartile range divided by the median, with the whiskers extending to a maximum of 1.5 times the interquartile range beyond the box. Outliers are shown as dots. Wilcoxon rank-sum tests; *P* values are two-sided. **h** Volcano plot showing differentially expressed genes between tumor clusters with top 10% quantile inflammatory scores vs. those with the bottom 10% quantile inflammatory scores (annotated in **f**). **i** Volcano plot showing differentially expressed genes between macrophages in tumors with top 10% quantile inflammatory tumor-cluster scores vs. macrophages in tumors with the bottom 10% quantile inflammatory tumor-cluster scores (annotated in **f**). **j** Kaplan–Meier survival analysis showing overall survival after initial pathological diagnosis. Statistical evaluation was performed using a two-sided log-rank test. Patients with high tumor-cell-intrinsic inflammation score (*n* = 26, top 25% percentile) displayed significantly lower chance of survival compared to patients with low inflammation score (*n* = 26, bottom 25% percentile) using bulk RNA-seq data. In **h** and **i**, statistical evaluation was performed using a two-sided Wilcoxon rank-sum test, applying Bonferroni correction for the resulting *P*-values. Source data are provided as a Source data file.

To search for intrapatient heterogeneity of tumor-cell transcriptomes, we identified 90 total clusters of tumor cells (over 50 cells per cluster) among the 30 tumor samples, with substantial inter-cluster transcriptional differences (“Methods”). Each sample averaged 3 tumor-cell clusters (Fig. 4a; examples in Fig. 4b). We sought to assess whether these transcriptome-based tumor subclusters represented genomically distinct subclones by systematically evaluating copy number variations (CNVs) across tumor subclusters inferred from the snRNA-seq data (Supplementary Fig. 4a). Specifically, we focused on known or candidate pathogenic targets in the regions with frequent arm-level and focal CNV events reported by the previous studies^12,13,58–61^. Some patients showed similar copy number profiles between tumor clusters, while others showed dramatically different copy numbers (Supplementary Fig. 4a).

One interesting example is patient C3N-01200, who displayed potentially genomically distinct tumor subclusters among the three tumor pieces profiled by snRNA-seq. The T1 piece (grade 2) harbored four major tumor subclusters (>50 cells), designated C1, C2, C3, and C4 (Fig. 4c). Most C1 and C4 cells showed copy number loss in chromosome 3p genes (*VHL*, *SETD2*), but no gains in chromosome 5q genes (*SQSTM1*, *RACK1*). C2 tumor cells showed both 3p loss and 5q gain. In contrast, C3 cells predominantly showed only 5q gain, not 3p loss. Tumor clusters from the other two tumor pieces (C3N-01200-T2 and C3N-01200-T3; both grade 4) also mostly showed 5q gain, but not 3p loss, similar to C3 from C3N-01200-T1.

Integration of tumor clusters mentioned above reveals that tumor cells of C3 from C3N-01200-T1 co-clustered with C3N-01200-T2 and C3N-01200-T3 tumor cells (Fig. 4d), which are primarily associated with 5q gain, not 3p loss (Fig. 4e). C2 cells from the T1 tumor piece, which showed 3p loss and 5q gain, remained a relatively distinct cluster in the integrated data. In this case, the tumor cells having only 5q gain but not 3p loss (C3 of T1, C1 and C2 of T2, and C1 of T3), the tumor cells with 3p loss but not 5q gain (C1 and C4 of T1), and the tumor cells with 3p loss and 5q gain (C2 of T1) may represent genetically distinct subclones. Additional experimental validation is needed to confirm the co-existence of tumor subpopulations with these specific CNV statuses as well as their transcriptome differences.

### Intratumor signaling heterogeneity revealed by single-cell tumor subclustering

For those genes and pathways most differentially expressed among intrapatient tumor subclusters, we performed an unbiased search across the MSigDB “Hallmark” gene sets (“Methods”). We found 38 sets differentially expressed among intrapatient tumor clusters relating to proliferation, immune, DNA damage, metabolism, development, and other signaling pathways (Fig. 4f). The most frequently differentially expressed gene sets include the proliferation pathways (mitotic spindle, E2F targets, and G2M checkpoint), genes downregulated in UV response, allograft rejection, and the epithelial–mesenchymal transition (EMT) pathways. Some pathways, like EMT, might be the first steps for tumor metastasis^62,63^, while others, such as the proliferation and DNA damage repair pathways, are important drivers for cancer progression^64,65^. Their differential representations between tumor cells within the same patients may indicate key roles in producing and amplifying tumor heterogeneity and driving clonal evolution during ccRCC progression.

To prioritize the gene sets important for tumor progression, we scored the above 38 gene sets across tumor clusters (Fig. 4f) and identified seven gene sets that are significantly associated with higher tumor stage (FDR < 0.1), namely inflammatory response (Fig. 4g), unfolded protein response, TGF-β signaling, TNF-α signaling via NF-κB, IL2-STAT5 signaling, IL6-JAK-STAT3 signaling, and apical junction. The tumor subclusters expressing high inflammation scores resemble cancer-cell-intrinsic inflammation. In this process, cancer cells alter the immune landscape by secreting inflammation-related cytokines or chemokines and subsequently promote cancer progression and metastasis^66^. We observed an upregulation of β2-microglobulin (B2M) expression in those cancer-cell-intrinsic inflammatory tumor subclusters (clusters with top inflammation score) vs. those with the lowest scores (log_2_FC = 0.91; Fig. 2h). B2M is a component of the MHC class I complex, and B2M expression on tumor cells protects them from phagocytosis^67^. This protection is mediated by the inhibitory receptor LILRB1, which is most highly expressed in the macrophages in our snRNA-seq dataset (Supplementary Fig. 4b). Macrophages in tumors with the top inflammatory response scores showed an upregulation of LILRB1 expression (log_2_FC = 0.89; Fig. 4i). These results suggest that inflammatory tumor subclusters might suppress macrophages through the B2M-LILRB1 interaction. In addition, cancer-cell-intrinsic inflammatory tumor subclusters also showed an upregulation of C1R (log_2_FC = 0.86), C1S (log_2_FC = 0.46), and C3 (log_2_FC = 0.48), while the corresponding macrophages showed an upregulation of C1q genes (*C1QA*, *C1QB*, *C1QC*). Based on previous studies^68,69^, the potential interaction between C1R and C1q we identified may indicate tumor-cell hijacking of macrophage-produced C1q to promote tumor growth. Finally, cancer-cell-intrinsic inflammatory tumor subclusters also showed an upregulation of the ectonucleotidase CD39 (*ENTPD1*; log_2_FC = 0.47), which is associated with resistance to immune-checkpoint blockade^70^ and worse prognosis^71^ in ccRCC.

Surveying the microenvironment of tumors with cancer-cell-intrinsic inflammatory tumor clusters, we observed a moderate association between tumor-cluster inflammatory score and macrophage content (R = 0.41, FDR = 0.11) and a significant upregulation of PDL2 (*PDCD1LG2*; log_2_FC = 0.19) in tumors with top inflammatory scores (Fig. 4i). We also observed a significant upregulation of PDL1 (*CD274*; log_2_FC = 1.0) and PDL2 (log_2_FC = 1.25) in classical dendritic cells (cDC) in tumors having top inflammatory scores. Of note, macrophages and cDCs account for 18.1% and 0.3% of cells in the tumor samples, respectively. Our findings suggest the activated inflammatory transcriptional program in ccRCC cancer cells might recruit and induce PDL2 + macrophages and PDL1/2+ cDCs, which in turn might inhibit T cell expansion and activity. Finally, we generated a cancer-cell-intrinsic inflammation gene signature by overlapping the tumor-cell-specific markers and the markers defining tumor clusters with the highest inflammatory score. The cancer-cell-intrinsic inflammation gene signature is associated with reduced overall survival in a larger patient cohort (*n* = 103) using bulk RNA-seq (Fig. 4j), suggesting this gene signature may characterize a more aggressive ccRCC. We also performed a similar analysis for the six other gene sets associated with higher tumor stages. Among them, TNF-α signaling via NF-κB and apical junction pathway signature scores are also significantly associated with worse overall survival (FDR < 0.05).

While previous studies based on human cells and mouse models showed cancer-cell-intrinsic inflammation was mainly caused by mutations in driver genes^66,72–74^, here we did not find any significant association between harboring top inflammatory tumor cluster and sample-level mutation in established ccRCC driver genes, such as *VHL*, *BAP1*, *PBRM1*, or *SETD2* (*P* > 0.3). However, we did observe that cluster-level copy number gain in chromosome 5q35.3 (includes *SQSTM1* and *RACK1*), as well as gain in 3q26.2 (includes *PRKCI* and *MECOM*), are positively associated with interferon-alpha response scores (FDR < 0.01), which in turn is also correlated with inflammatory response scores (Pearson’s r = 0.65, *P* < 0.05). One example is the case of patient C3N-1200. In this patient, the three tumor subclusters, including C1 and C2 from T2 and C1 from T3, all showed copy number gain in 5q and 3q genes (Fig. 4f) and high scores in interferon-alpha signaling and inflammatory response. Interestingly, these clusters, which account for 26.5% of the cells sampled from this patient, also showed outlier scores in EMT, proliferation-related, PI3K-AKT-MTOR signaling, and TGF-β signaling pathways, suggesting strong metastatic potential. Finally, we also identified meta-clusters, which represent shared variations in the tumor-cell transcriptome across patients. We found that 84% of the 38 gene sets differentially expressed among intrapatient tumor clusters are also significant distinguishing features of the meta-clusters, including inflammatory response and EMT pathways, supporting our analysis described above (Supplementary Fig. 5, Supplementary Notes).

### Tumor subgroups with distinct epithelial and mesenchymal features

**Fig. 5:**
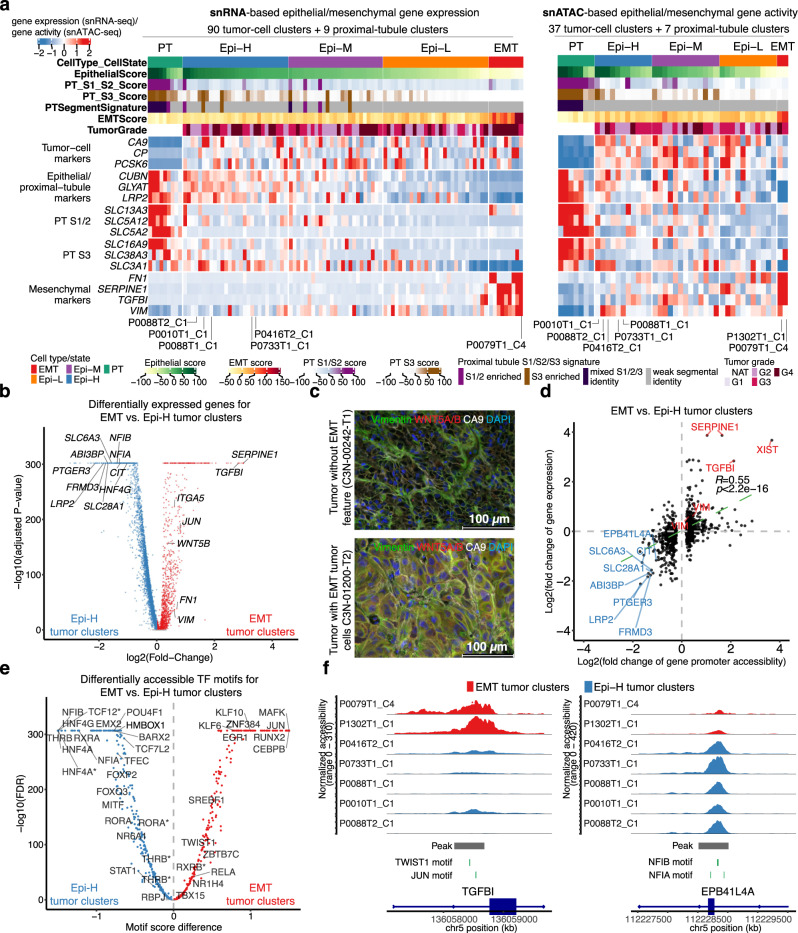
Four tumor subgroups with distinct epithelial and mesenchymal features. **a** Left: Heatmap showing gene expression of the epithelial and mesenchymal marker genes for tumor clusters and proximal tubule (PT) clusters (>50 cells) using snRNA-seq data. Right: Heatmap showing gene activity of the epithelial and mesenchymal marker genes for tumor clusters and PT clusters (>50 cells) using snATAC-seq data. **b** Volcano plot showing differentially expressed genes between the EMT tumor clusters and Epi-H tumor clusters highlighted in (**a**). Labels on the right denote known mesenchymal markers, while those on the left denote known markers for PT cells. Statistical evaluation was performed using a two-sided Wilcoxon rank-sum test, applying Bonferroni correction for the resulting *P*-values. **c** Immunofluorescence staining of vimentin (VIM), CA9, WNT5A/B, and DAPI, showing VIM and WNT5A/B in CA9 + cells in the cross-sections of the tumor with EMT tumor cells (C3N-01200-T2), but not in the control tumor (C3N-00242-T1). Two independent experiments were performed with similar results. Scale bar, 100 μm. **d** Scatter plot displaying the log2 transformed fold change for gene promoter accessibility versus log2 transformed fold change for gene expression in EMT tumor clusters vs. Epi-H tumor clusters (shown in **b**). The *P*-value is derived from a two-sided Spearman rank correlation test (*P*-value = 9.2e−72). **e** Volcano plot showing differentially accessible TF motifs between the EMT tumor clusters and Epi-H tumor clusters. Asterisks denote the var.2 version of the TF motif based on the JASPAR database. Statistical evaluation was performed using a two-sided Wilcoxon rank-sum test, applying Benjamini–Hochberg correction for the resulting *P*-values. **f** Genomic regions near *TGFBI* (upregulated in EMT tumor clusters) and *EPB41L4A* (upregulated in Epi-H tumor clusters). The plots show the normalized accessibility by snATAC-seq around these regions in EMT tumor clusters (red) and Epi-H tumor clusters (blue). Source data are provided as a Source data file.

The EMT pathway, which can increase tumor-initiating and metastatic potential for tumor cells^75,76^, is one of the top differentially expressed gene sets among the intrapatient tumor-cell clusters. Furthermore, high EMT scores were associated with advanced tumor stages (Fig. 4g; *P* < 0.05, FDR = 0.11), suggesting higher EMT pathway expression in tumor subclusters is indicative of RCC progression. To better understand and characterize EMT in ccRCC progression, we assembled a panel of markers to calculate mesenchymal and epithelial feature scores for 90 tumor-cell clusters and 9 proximal-tubule clusters (Fig. 5a, “Methods”, Supplementary Data 4). We identified 4 major tumor subgroups, including three subgroups with strong, medium, and low epithelial features, all of which have relatively low mesenchymal features (denoted as Epi-H, Epi-M, and Epi-L tumor clusters, respectively); and one subgroup with outlier mesenchymal feature scores (denoted as EMT tumor clusters). The continuum of epithelial and mesenchymal features across tumor clusters was further supported by the chromatin-accessibility-based gene activities derived from snATAC-seq data. Specifically, five Epi-H tumor clusters with high epithelial gene expression scores showed high epithelial gene activity scores, and two EMT tumor clusters also showed similar mesenchymal gene activity scores (Fig. 5a). Regardless of the epithelial/mesenchymal feature scores, the tumor subgroups overall showed higher expression of tumor-cell markers, such as *CA9*, *CP*, and *PCSK6*, while PT clusters overall showed higher proximal tubule markers, such as *CUBN*, *GLYAT*, and *LRP2*.

To further identify genes that characterize the tumor subgroups above, we compared gene expression profiles between the EMT tumor clusters and the Epi-H tumor clusters that were supported by both snRNA-seq and snATAC-seq data. We detected many known EMT regulators upregulated in the EMT tumor population (Fig. 5b), such as *SERPINE1*^77^, *TGFBI*^78^, *WNT5B*^79^, vimentin^80^ (*VIM*), and fibronectin^81^ (*FN1*). These upregulated genes indicate that the EMT population possesses strong mesenchymal potential and may represent a pre-metastatic tumor population. In addition, we validated vimentin and WNT5B using immunofluorescence staining in a tumor with EMT tumor cells compared to another tumor without EMT tumor cells (Fig. 5c).

We hypothesized that key genes defining the epithelial–mesenchymal scores are epigenetically regulated due to the correlation between the upregulation of these genes and their promoter accessibility. Indeed, the gene expression changes between the two tumor groups showed a significant positive correlation with their promoter accessibility changes. *SERPINE1* and *TGFBI* had the highest increased promoter accessibility and gene expression in EMT tumor clusters (fold change >2). 8 genes, namely *LRP2*, *EPB41L4A*, *SLC6A3*, *FRMD3*, *PTGER3*, *ABI3BP*, *SLC28A1*, and *CIT* showed over 2-fold changes in increased promoter accessibility and expression in Epi-H tumor clusters (Fig. 5d). To understand which TFs may regulate the transcription of the above genes, we compared the TF binding accessibilities between the EMT tumor clusters and the Epi-H tumor cluster, prioritizing TFs that were differentially expressed between the two groups (Fig. 5e). The EMT tumor population showed increased binding accessibility for known positive regulators of EMT, such as TWIST1 and JUN (Fig. 5e, Supplementary Data 4). We also observed increased motif accessibility for the hepatocyte nuclear factors (HNF4A and HNF4G) in the Epi-H tumor clusters. These transcription factors are known to regulate kidney development^82,83^. To connect these differentially enriched TFs to the differentially expressed genes, we subsequently searched for the binding motifs of these TFs in the promoter regions of these genes. One example is *TGFBI*, which showed increased promoter accessibility and gene expression in the two EMT tumor clusters. The *TGFBI* open promoter region harbors motifs for TWIST1 and JUN, consistent with the reported roles of these TFs regulating *TGFBI* transcription^84,85^. Conversely, *EPB41L4A* showed increased promoter accessibility and gene expression in the Epi-H tumor clusters. Taken together, these data indicate that many genes distinguish tumor groups with distinct epithelial and mesenchymal features, such as *WNT5B*, as well as *EPB41L4A* and *TGFBI*, controlled epigenetically by an array of transcriptional factors delineated above and chromatin accessibility changes.

### Chromatin accessibility changes in *BAP1* and *PBRM1* mutant tumors

We next sought to understand the expression signatures of tumors harboring *BAP1* and *PBRM1* mutations and the impact of these mutations on chromatin accessibility that may underpin such expression signatures. For snATAC-seq analysis, we selected 4 *BAP1*-mutant tumors, 9 *PBRM1*-mutant tumors, 2 tumors with both *BAP1* and *PBRM1* mutations, and 8 tumors without mutations in either *PBRM1* or *BAP1* (Supplementary Fig. 6a). All of these samples have matching snRNA-seq data, and almost all mutant samples carry the *VHL* mutation (all except for one with *VHL* promoter hypermethylation) and 3p loss opposite to the mutated alleles (Supplementary Figs. 1a, 6a).

**Fig. 6:**
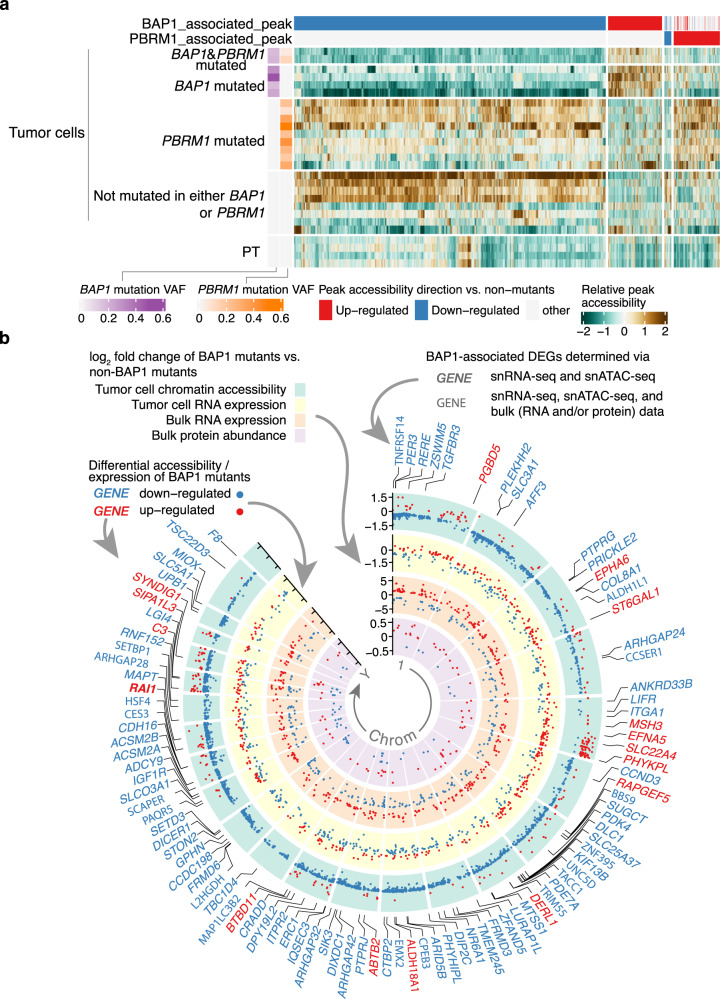
Chromatin accessibility landscape of BAP1 and PBRM1 mutant tumors. **a** Heatmap showing the relative changes in ATAC-peak accessibility for peaks differentially accessible between the tumor cells of *BAP1*-mutated tumors (6 tumors, including 2 *BAP1*- and *PBRM1*-mutated tumors, 29,366 cells) vs. non-*BAP1/PBRM1*-mutated tumors (8 tumors; non-mutants) and peaks differentially accessible between tumor cells of *PBRM1*-mutated tumors (9 tumors, 32,255 cells) vs. non-*BAP1/PBRM1*-mutated tumors (non-mutants). Each column is an ATAC peak, and only significantly and consistently changed peaks are plotted (FDR < 0.05, “Methods”). Only samples with >8% mutation VAF in *BAP1* or *PBRM1* are shown (1 sample excluded). **b** Circos plot showing the genome-wide chromatin accessibility, gene expression, and protein abundance changes associated with *BAP1* mutation. The green circle contains significantly different ATAC peaks in *BAP1*-mutated vs. non-*BAP1*-mutated tumors, with each dot representing one ATAC peak. The labeled y-axis represents the fold changes of the peak accessibility change. Red and blue dots denote peaks with higher and lower accessibility peaks in *BAP1*-mutated vs. non-*BAP1*-mutated tumors, respectively. The yellow circle plots the fold changes of the differentially expressed genes (DEGs) associated with *BAP1* mutation discovered by snRNA-seq data (FDR < 0.05), with each dot representing one gene. The orange circle displays the fold changes of the DEGs associated with *BAP1* mutation (FDR < 1e−04, |log_2_FC| > 1) discovered by the CPTAC bulk RNA-seq data (*n* = 103). The innermost purple circle plots the fold changes of differentially expressed proteins associated with *BAP1* mutation (FDR < 0.05) discovered by the CPTAC bulk proteomics data (*n* = 103). Similarly, the red and blue colors for the dots denote higher and lower expression for the genes/proteins in *BAP1*-mutated vs. non-*BAP1*-mutated tumors. The gene symbols highlighted outside the circles represent genes showing the consistent direction of snRNA-seq and snATAC-seq changes in *BAP1* mutants vs. non-mutants (absolute log2 fold change >= 0.3). Red gene symbols represent genes with increased promoter/enhancer accessibility and gene expression in *BAP1* mutants. Blue gene symbols represent genes with decreased promoter/enhancer accessibility and gene expression in *BAP1* mutants. Gene symbols in bold font represent the genes mentioned above with consistent expression change in either bulk RNA-seq or bulk protein data. Source data are provided as a Source data file.

To understand the impact of *BAP1* deficiency on chromatin accessibility, we used snATAC-seq data to analyze differentially accessible chromatin regions (DACRs) by comparing the tumor cells of *BAP1*-mutants versus tumor cells from tumors without *PBRM1* or *BAP1* mutations. We identified 4554 such DACRs. Interestingly, most of these regions (84%, 3829 peaks) showed reduced accessibility in *BAP1-*mutants (Fig. 6a, Supplementary Data 5), which is consistent with previous reports that *BAP1* loss induces chromatin condensation^23,24^. We also analyzed DACRs for *PBRM1* mutants (9 tumors, not including 2 with both *BAP1* and *PBRM1* mutations) versus tumors without *PBRM1* or *BAP1* mutations (again using only tumor cells). We identified 646 DACRs, with the majority (87%, 561 DACRs) having increased accessibility in *PBRM1*-mutants (Fig. 6a, Supplementary Data 5). Moreover, *BAP1* mutation seems to have a dominant effect compared to *PBRM1* mutation, as the two tumors with both *BAP1* and *PBRM1* mutations showed more similar patterns in chromatin accessibility to *BAP1*-only mutated tumors (mean Pearson’s r = 0.47) than the *PBRM1*-only mutated tumors (mean Pearson’s r = −0.12; Fig. 6a). Through these analyses of snATAC-seq data, we observed that *BAP1*-deficient tumors undergo more global changes in chromatin accessibility compared to the *PBRM1*-deficient tumors in ccRCC.

### Impact of *BAP1* mutations on the transcriptional network in ccRCC

**Fig. 7:**
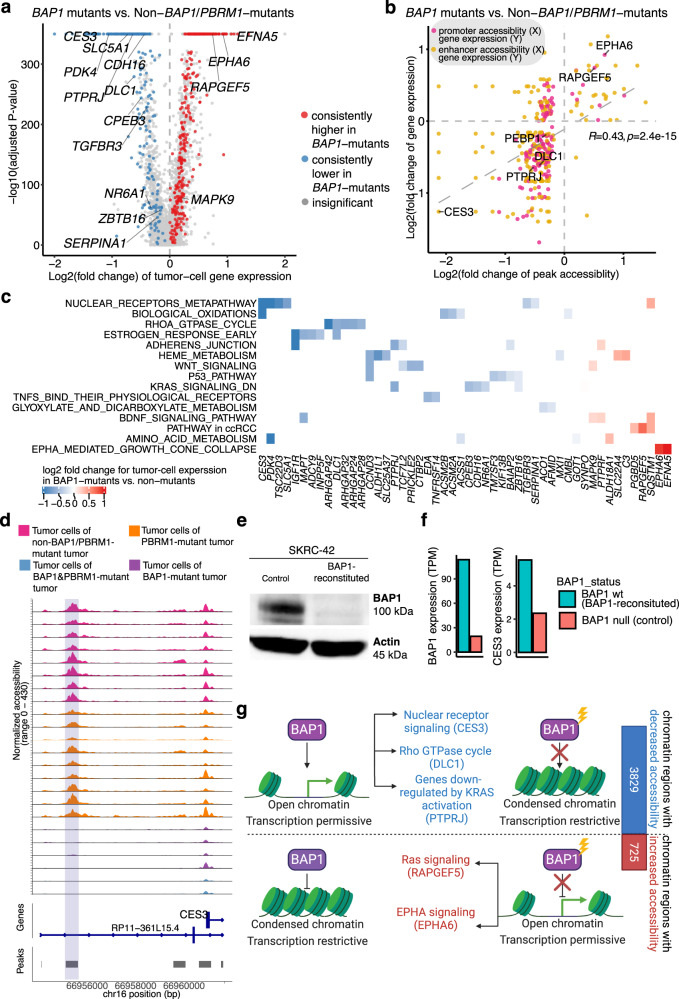
Impact of BAP1 mutations on chromatin accessibility and transcriptional networks. **a** Volcano plot displaying the differentially expressed genes (DEGs) between the tumor cells of *BAP1*-mutated tumors (26,806 cells) vs. tumor cells of non-*BAP1/PBRM1*-mutated tumors (31,002 cells) by snRNA-seq data. Statistical evaluation was performed using a two-sided Wilcoxon rank-sum test, applying Bonferroni correction for the resulting *P*-values. Dots are colored by whether the genes showed significant and consistent fold changes in individual comparisons of each *BAP1*-mutated tumor vs. non-*BAP1/PBRM1*-mutated tumors. **b** Scatter plot showing the positive correlation of chromatin accessibility and transcriptional changes. The log_2_(fold change) of the snRNA-seq expression for each gene (mRNA) is plotted against the log_2_(fold change) in the relative snATAC-seq peaks (for all the genes or promoter/enhancer peaks with significant fold change in over 50% of the comparisons for individual *BAP1*-mutated tumor vs. non-*BAP1/PBRM1*-mutated tumors). The *P*-value is derived from a two-sided Spearman rank correlation test (*P*-value = 2.4e−15). Each dot represents a gene-peak pair. Dots are colored by whether the peak overlaps the gene promoter or is a potential enhancer (co-accessible with the promoter peak). **c** Heatmap showing the pathways associated with the BAP1-associated DEGs with promoter/enhancer accessibility change (represented in **b**). **d** Genomic regions near the *CES3* gene in BAP1-mutated tumors vs. non-*BAP1*-mutated tumors. The plots show the normalized accessibility signal by snATAC-seq around these regions in tumor cells of *BAP1*-mutant tumors (purple), tumor cells of *PBRM1*-mutant tumors (orange), tumor cells of non-*BAP1*/*PBRM1*-mutant tumors (pink). **e** Western blot showing BAP1 and Actin protein levels in the BAP1-reconstituted and control SKRC-42 cells. Three independent experiments were performed that showed similar results. **f** Bar plot showing *BAP1* and *CES3* gene expression in the BAP1-reconstituted and control SKRC-42 cells. **g** Schematic diagram showing the differential effects of *BAP1* mutations on chromatin accessibility. Created with BioRender.com. Source data are provided as a Source data file.

*BAP1*-associated DACRs with decreased accessibility are distributed across all chromosomes, with hotspots in chromosomes 11 and 19 (Supplementary Fig. 6b), overlapping genes such as *DIXDC1* and *LGI4* (Fig. 6b). DACRs with increased accessibility in *BAP1*-mutants were more sparsely distributed, with hotspots in chromosome 5 (Supplementary Fig. 6b). We further focused our analysis on those genes’ expression changes associated with *BAP1* mutations that could be linked to the changes in DNA accessibility. We compared the tumor-cell expression profiles of *BAP1*-mutant tumors versus non-*BAP1* and non-*PBRM1*-mutant tumors using snRNA-seq data and identified 563 differentially expressed genes (Fig. 7a; Supplementary Data 5). As expected, the changes in gene expression and associated promoter/enhancer peak accessibility are significantly positively correlated (Fig. 7b). Figure 7c highlighted some of the genes with both decreased accessibility and expression in *BAP1* mutants, which were most enriched in the nuclear receptor meta-pathway (*CES3*, *PDK4*, *SERPINA1*, *SLC5A1*, and *TGFBR3*), the RhoA GTPase cycle (*DLC1*, *ARHGAP24/28/32/42*), and genes downregulated by KRAS activation (*PTPRJ*, *CDH16*, *CPEB3*, *NR6A1*, and *ZBTB16*), among others. Genes with increased accessibility and expression in *BAP1* mutants include known ccRCC-associated genes (*RAPGEF5* and *SQSTM1*) and EPHA signaling genes (*EPHA6* and *EFNA5*). We also performed a similar analysis for *PBRM1* mutants (Supplementary Fig. 6c).

We used the larger bulk gene expression and protein datasets (*n* = 103) to prioritize identified *BAP1*-specific DEGs (bold gene symbols in Fig. 6b). We found that 224 DEGs were consistently down/upregulated in bulk gene expression and that 21 of them also showed consistent patterns in bulk protein data (Supplementary Data 5). One of the most striking examples from this analysis was *CES3*, which showed both reduced gene expression and reduced accessibilities of associated enhancer peak in *BAP1*-mutant tumor cells (Fig. 7b). We identified a potential *CES3* enhancer peak located ~5 kb upstream of the *CES3* transcriptional start site (TSS), displaying consistently lower accessibility in tumor cells of all *BAP1* mutants as compared with other tumors (Fig. 7d) and PT cells from NATs (Supplementary Fig. 6d). We found that reduced *CES3* DNA accessibility and gene expression were associated with *BAP1* mutations, supported by both bulk RNA and protein data. *CES3* encodes a carboxylesterase with crucial roles in xenobiotic metabolism. *CES3* down-regulation affects lipid metabolism^86^ and might promote tumor progression in *BAP1*-mutants. We utilized a pair of isogenic ccRCC cell lines derived from SKRC-42 (BAP1 null) to validate our observation regarding *CES3*. First, we confirmed the BAP1 status of these lines using a western blot (Fig. 7e). Next, we found that *CES3* expression in the SKRC-42-control (BAP1 null) is 42% of that in the BAP1-reconstituted derivative in our bulk RNA-seq data (Fig. 7f), supporting our observation from snRNA-seq data.

Together, we developed a working model to illustrate the effects of *BAP1* mutations on ccRCC transcriptome through chromatin accessibility changes (Fig. 7g). Compared to *PBRM1* mutations, *BAP1* mutations seem to exert moderate but more widespread effects on chromatin accessibility. The predominant *BAP1* mutation effect on chromatin appears to be decreasing chromatin accessibility. In contrast, *PBRM1* mutation is mainly associated with increased chromatin accessibility (Fig. 6a). Furthermore, we observed many genes downregulated in *BAP1*-mutants could be linked to the widespread decreased chromatin accessibility associated with *BAP1* mutations (Figs. 6b, 7b, c), and experimentally validated one of them being the *CES3* gene.

## Discussion

This report describes findings of a combined application of snRNA-seq and snATAC-seq in ccRCC to study transcriptional profiles and chromatin accessibility patterns at the single-nucleus level. We identified 324 tumor-cell-specific markers compared to other cell types, a majority of which were supported by other published ccRCC single-cell datasets (Supplementary Notes). Of the 20 prioritized markers, four have been associated with ccRCC but not evaluated in a single-cell context (*ABCC3*^87^, *KCTD3*^88^, *SEMA6A*^89^, *PLEKHA1*^90^) and nine are known tumor markers of ccRCC (*TGFA*^91^, *PLIN2*^92^, *FTO*^93^, *SLC6A3*^94^, *NDRG1*^95^, *CP*^96^, *EGFR*^97^, *ENPP3*^98^, *COL23A1*^99^). Seven markers (*SNAP25*, *PHKA2*, *EPHA6*, *ABLIM3*, *SHISA9, PCSK6*, *UBE2D2)* do not have well-defined functions in ccRCC. Even in cases where these genes have been previously identified, the added value of current technologies is that we have been able to compare the expression of these markers in tumor cells to non-tumor cell types; our analysis supports these genes as tumor-cell-specific markers for ccRCC.

We chose to focus on ceruloplasmin (*CP*) because it predicted worse survival and displayed an interesting spatial expression pattern associated with hyalinized stroma. Known *CP* functions mainly involve copper transport, ferroxidase activity, angiogenesis, and regulating oxidative stress^100^. Bulk and scRNA-seq studies showed that *CP* is overexpressed in ccRCC compared to normal adjacent tissue^31,37,38^ and other RCC subtypes^101^. Several studies showed associations between *CP* and higher grades and poor prognosis in ccRCC^36,96^, and one study suggested that *CP* knockdown impaired the cell invasion capability of RCC cells^38^. To date, few studies have undertaken functional investigations of *CP* in ccRCC. Here, we observed that EMT pathway genes, including *CD44* and *TGFBI*, were downregulated after *CP* knockdown (Supplemental Data 3), suggesting *CP* is required for cell invasion. We also observed a down-regulation of inflammatory response genes associated with *CP* knockdown (Fig. 2d), consistent with reports of *CP*’s role in inflammation. *CP* is a HIF1A target and helps stabilize the HIF1A protein^102,103^. Indeed, our study shows that hypoxia response genes were enriched in genes downregulated after *CP* knockdown, including *IGFBP3* and *ANGPTL4* (Supplemental Data 3). Furthermore, we revealed the spatial heterogeneity of *CP* using spatial transcriptomics and *CP*’s potential role in mediating tumor-stroma interactions in ccRCC with the snRNA-seq data of ccRCC tumor tissue and RNA-seq data of cells in which *CP* was suppressed by shRNA. We also found that the KLF9 transcription factor may regulate *CP* transcription. Together, these results further our understanding of *CP* in ccRCC^96^.

We also demonstrate the utility of considering epigenetic regulation in understanding the cell origin of cancer cells. For example, while previous studies relied on mutational and transcriptional similarities in support of PT as the cell of origin for ccRCC^44,104–106^, we find strong epigenetic evidence suggesting this as well. When we compared chromatin accessibility patterns between tumor cells and normal PT cells, we uncovered many tumor-cell-specific TFs beyond the well-known TF – HIF1A: MXI1, KLF9, RBPJ, and NFKB1/2 have been previously implicated in renal cancer tumorigenesis^107–112^; and HSF2 and SREBF2 were linked to renal tubular cell injury^113,114^. As expected^115^, we observed a dozen genes upregulated in the glycolysis pathway in ccRCC cells compared to normal PT cells. Studies have shown that transcription factors, such as HIF-1, c-MYC, p53^50^, and SIX1^51^ play direct roles in regulating aerobic glycolysis. We report several TFs that may regulate glycolytic genes in ccRCC, particularly MXI1 and KLF9. MXI1, a member of the Mad family proteins known to antagonize c-MYC-dependent transcription, has a potential oncogenic role in RCC, as MXI1 knockdown impaired kidney cancer xenograft formation in nude mice^107^. Our results suggest that MXI1 promotes ccRCC cell growth by activating the transcription of hexokinase 2 (HK2). KLF9 is a Krupple-like transcription factor (KLF) and was shown to transactivate *KCNQ1*^110^ and *SNX5*^109^, inhibiting ccRCC cell proliferation. In snRNA-seq data, KLF9 is not significantly differentially expressed between ccRCC tumor cells vs. PT cells. Nonetheless, KLF9 knockdown is associated with a concomitant upregulation of many glycolytic genes, such as *HK2*, *PFKP*, and *ENO2*. Our results warrant more experimental investigations to validate the transcriptional regulation and test whether KLF9 affects the metabolic flux.

In evaluating tumor cell heterogeneity, many patients exhibited 3–4 distinct tumor-cell clusters. We acknowledge that the number of clusters per cancer may be influenced by the analysis parameters and subdivisions of existing clusters can be achieved without the samples changing their intrinsic heterogeneity. We identified cases where tumor subclusters display distinct copy number statuses (inferred based on snRNA-seq data). Several clusters from one patient, C3N-01200, showed almost no 3p loss and only 5q gain, both frequent alterations in ccRCC^12,116^. Our observations suggest two possibilities: (1) some ccRCC cells may initiate by 5q gain; (2) some ccRCC cells may initiate by silencing the 3p genes through non-CNV mechanisms (such as mutations) and co-exist with other subclones who initiate by 3p copy loss. Future studies using single-cell WES may be able to provide additional insights since mutation mapping by snRNA-seq was sparse (Supplementary Fig. 7).

We identified four distinct tumor subgroups in terms of epithelial and mesenchymal features. Epithelial tumor clusters are sporadic concerning the traditional PT S1/2 and S3 group classification markers, suggesting these tumor cells might not come from one group of S1/2 or S3 proximal tubule cells. We additionally observed *WNT5B* upregulation in the ccRCC EMT tumor subpopulation. WNT5B is a member of the WNT5 protein subfamily and signals through the non-canonical beta-catenin-independent pathway^117^. It is required for cell migration, proliferation, and differentiation in many cell types^117^ and has an emerging role in mediating EMT and cell migration in breast, pancreatic, and colorectal cancers^118–120^. However, the role of *WNT5B* in ccRCC is unclear. We showed *WNT5B* upregulation in ccRCC tumor subpopulations with EMT features. We validated the WNT5B protein expression in a patient tumor with the EMT signature population (Fig. 5c), suggesting WNT5B might mediate the EMT process in ccRCC.

*BAP1* and *PBRM1* mutations are associated with distinct overall survival, leading to the first molecular classification of sporadic ccRCC^18,19^. However, we knew little about the epigenetic alterations brought upon by *BAP1* and *PBRM1* mutation leading to the distinct phenotypes in ccRCC. Our snATAC-seq analysis revealed a large portion of genomic loci displaying decreased accessibility in ccRCC with *BAP1* mutations, consistent with the role of BAP1 in the global chromatin condensation and transcriptional activation reported in non-RCC contexts^121^. The presence of both increases and decreases in chromatin accessibility suggests that the BAP1-mediated regulation of chromatin accessibility depends on the epigenetic landscape. In addition, we delineated genes whose expression levels are affected by *BAP1* mutation and could be attributed to their altered chromatin accessibility, validating one such gene, *CES3*. On the other hand, *PBRM1* encodes BAF180, a subunit of nucleosome remodeling complex PBAF. In contrast to the *BAP1* mutation, *PBRM1* mutation was primarily associated with ATAC-peaks exhibiting increased accessibility, suggesting PBRM1 may have a role in gene silencing, consistent with a previous report using osteosarcoma cells^25^. These results provide hypotheses for future functional studies of *BAP1* and *PBRM1* in ccRCC.

Our study provides a resource of single-nucleus epigenomic, transcriptomic, and spatial transcriptomics data where we explored heterogeneous signaling activities and epigenetic regulation of tumor subpopulations in ccRCC. Compared to single-cell RNA-seq, snRNA-seq can be used to analyze archival specimens and minimize stress responses from cell dissociation necessary for scRNA-seq. It should be noted that the single-nucleus approach generally does not cover transcripts from the small mitochondrial genome. Unlike scRNA-seq surveying the total RNA in a cell, snRNA-seq captures the nuclear RNA, closely reflecting current transcriptional regulation in the cell.

In summary, our comprehensive integrative analyses of a broad range of multi-omics data, especially with the inclusion of epigenetic and spatial omics results, allowed us to identify ccRCC tumor markers valuable for diagnosis, prognosis, and development of future therapies, including CAR-T therapy. In the case of known tumor marker *CP*, while *CP* knockdown in ccRCC has been done previously^38^, our experimental data here provided specific candidate genes potentially regulated by *CP*, such as *COL4A1* and *OSMR*, that warrant follow-up studies to understand *CP*’s function. As for the transcriptional regulation of *CP*, future studies using ChIP-seq and 3C experiments may provide direct evidence for TFs that bind and regulate *CP* expression. Similarly, the chromatin accessibility changes associated with the chromatin modifiers BAP1 and PBRM1 described in this manuscript will benefit from additional epigenetic assays such as ChIP-seq and transposase-directed transposon insertion mapping to ascertain the direct effect of BAP1 and PBRM1 on the candidate affected genes. Finally, in addition to enhancing our understanding of the molecular features and the oncogenic mechanism of ccRCC, we hope the discoveries described above and the large volume of data generated will empower a wide range of follow-up studies in the RCC community and beyond.

## Methods

### Human tissue specimens and clinical data

We obtained 34 specimens (used in the CPTAC ccRCC discovery study^13^) from the CPTAC Biospecimen Core Resource to perform snRNA-seq and snATAC-seq experiments. Institutional Review Boards (IRBs) of Spectrum Health Services, University of Pittsburgh IRB, Beaumont Health Biobank, International Institute for Molecular Oncology, BioPartners and Asterand Bioscience reviewed protocols and consent documentation, in adherence to the CPTAC guidelines. Informed written consent was obtained from all participants for sharing individual-level data. We selected 34 samples from this corpus, with a balanced representation of mutation status, immune subtypes, and druggable events. More specifically, we selected comparable numbers of samples with *PBRM1* and *BAP1* mutations alone and samples without mutations in either of these genes, samples with immune inflamed and immune dessert subtypes, and samples with c-MET overexpression, the last being a promising druggable target in the lab. Finally, we requested the remaining cryo-pulverized tissue (the very same pool of tissue powder that was used for the original bulk sequencing) for single nuclei RNA-seq (snRNA-seq) and single nuclei ATAC-seq (snATAC-seq). Additional tumor segments were selected by the CPTAC Biospecimen Core Resource based on availability. Additional tumor segments processed for snRNA-seq were selected based on the successes of the original tumor segment snRNA-seq, the weight of additional tumor samples, and mutation status (the four cases selected had different *PBRM1* and *BAP1* mutation statuses). Demographics, histopathologic information, and treatment details were collected by the CPTAC consortium and were retrieved via the CPTAC Data Portal at: https://cptac-data-portal.georgetown.edu/study-summary/S044. Self-reported gender information was collected by CPTAC. Altogether, we have 18 male and 7 female participants based on self-reported gender information. The age distribution is 30–49 (16%), 50–69 (68%), and 70–74 (16%). None of the study participants was compensated.

### Cell lysis

15–25 mg of pulverized tissue was placed in a 5 mL Eppendorf tube on ice. Using a wide-bore pipette tip (Rainin), a lysis buffer prepared from the Nuclei Isolation protocol (10x Genomics) and SuperRNase inhibitor (Invitrogen) was added to the tube. The tissue solution was gently pipetted until the lysis liquid turned a slightly cloudy color. (The number of pipetting iterations depended on the specific tissue.) The tissue homogenate was then filtered through a 40-micron strainer (pluriSelect) and washed with a BSA wash buffer (2% BSA + 1× PBS + RNase inhibitor). The filtrate was collected, centrifuged at 500 × *g* for 6 min at 4 °C, and resuspended with a BSA wash buffer.

### Fluorescence-activated cell sorting (FACS)

100 µL of cell lysis solution was set aside for unstained reference, while the rest was stained with DRAQ5 or 7AAD for RNA or ATAC sequencing, respectively. Namely, snRNA-seq nuclei were stained with 1 µL of DRAQ5 per 300 µL of the sample, and snATAC-seq nuclei were stained with 1 µL of 7AAD per 500 µL of the sample. Sorting gates were based on size, granularity, and dye staining signal (Supplementary Fig. 8).

### 10x library preparation and sequencing of snRNA-seq and snATAC-seq

Nuclei and barcoded beads were isolated in oil droplets via the 10x Genomics Chromium instrument. Single nuclei suspensions were counted and adjusted to a range of 500 to 1800 nuclei/µL using a hemocytometer. Reverse transcription was subsequently performed to incorporate cell and transcript-specific barcodes. All snRNA-seq samples were run using the Chromium Next GEM Single Cell 3’ Library and Gel Bead Kit v3.1 (10x Genomics). For snATAC-seq, Chromium Next GEM Single Cell ATAC Library and Gel Bead Kit v1.1 prep (10x Genomics) were used for all samples. Barcoded libraries were then pooled and sequenced on the Illumina NovaSeq 6000 system with specific flow cell types (snRNA-seq: S4; snATAC-seq: S1).

### Patient-derived xenograft tumor specimen collection and preparation

The tumor materials for the patient-derived xenograft (PDX) model were obtained from patients either via core needle biopsy or surgical resection after informed consent. All human tissues acquired for experiments were processed in compliance with NIH regulations and institutional guidelines, as approved by the Institutional Review Board at Washington University in St. Louis (WUSTL). All animal procedures were reviewed by and received ethical approval from the Institutional Animal Care and Use Committee (IACUC) at WUSTL. Our animal protocol sets the maximal tumor size at 2 cm diameter. In these studies, this limit has not been exceeded. For implantation, 6–8-week-old female immunodeficient NSG mice (Strain: NOD.Cg-*Prkdc*^*scid*^ *Il2rg*^*tm1Wjl*^*/SzJ*, Stock No: 005557) were purchased from The Jackson Laboratory. The sex assignment was done by the Jackson Laboratory. The sex of the animal was not considered in the study design as the study only focus on the characteristics of the human tumor, which was implanted in the immunodeficient NSG mice. Furthermore, only female mice were used. Thus, sex-based analyses for the mice were not performed. Mice were housed in a temperature-controlled facility (68–72 °F and 45–55% relative humidity) on a 12–12-h light-dark schedule with normal food and sterile water supplies. Anesthesia was given before tumor implantation subcutaneously on both flanks. Animals were euthanized using CO_2_ per NIH Institutional Animal Care and Use Committee (IACUC) guidelines and tumors were harvested from mice once they reached 2 cm in volume. 4% paraformaldehyde was used to fix the tumor at 4 °C and was processed the next day before embedding in paraffin wax; small pieces (3 mm cubes) were frozen in 10% dimethylsulfoxide (DMSO) (Sigma-Aldrich, D2660) and 90% fetal bovine serum (Gibco, 10437028) at –80 °C and later transferred to liquid nitrogen for future implantation.

### Immunofluorescence (IF) staining

5-micron thickness cut ccRCC Formalin-Fixed Paraffin-Embedded (FFPE) sections were deparaffinized and rehydrated using xylene, high to low percentages of ethanol, and finally placed in 1× PBS. The heat antigen retrieval method was applied using 1 mM EDTA for at least 25 min. 5% Donkey serum and 1% BSA was used as blocking buffer and as primary and secondary antibodies diluent. Antibodies for CA9 Rabbit (#NB100-417; Polyclonal; Novus Bio) at 1:350, CA9 Goat (#PA5-47268, Polyclonal; Invitrogen) at 1:50, VIM Chicken (#NB300-223; Polyclonal; Novus) at 1:150, WNT5a/b Rabbit (#55184-1-AP, Polyclonal; Proteintech) at 1:100, CP Goat (#A80-124A; Polyclonal; Bethyl lab) at 1:100, and PCSK6 Rabbit (#PA5-32966; Polyclonal; Invitrogen) at 1:100 were applied on sections and later detected with specific fluorescent secondary antibodies conjugated with Alexa Fluor 488 Donkey anti-Rabbit #711-546-152, Alexa Fluor 488 Donkey anti-Chicken #703-606-155, Alexa Fluor 488 Donkey anti-Goat #705-546-147, Alexa Fluor 594 Donkey anti-Rabbit #711-586-152, Alexa Fluor 594 Donkey anti-Goat #705-586-147, Alexa Fluor 647 Donkey anti-Goat #705-607-003 from Jackson ImmunoResearch diluted at 1:1000. All IF images were taken using a Lecia DMi8 fluorescence microscope.

### FFPE Spatial transcriptomics specimen collection and preparation

For spatial transcriptomics, the samples were collected with informed written consent in concordance with the Washington University Institutional Review Board (IRB) at the Washington University School of Medicine in St Louis (St Louis, MO). Primary clear cell renal cell carcinoma samples were collected during surgical resection and verified by standard pathology.

The RNA quality of FFPE tissue blocks was evaluated by calculating DV200 of RNA extracted from FFPE tissue sections following the Qiagen RNeasy FFPE Kit protocol. After the Tissue Adhesion Test, 5 μm sections were placed on the Visium Spatial Gene Expression Slide following Visium Spatial Protocols-Tissue Preparation Guide (10x Genomics, CG000408 Rev A). After overnight drying, slides were incubated at 60 °C for 2 h. Deparaffinization was then performed following Visium Spatial for FFPE—Deparaffinization, H&E Staining, Imaging & Decrosslinking Protocol (10x Genomics, CG000409 Rev A). Sections were stained with hematoxylin and eosin and imaged at 20x magnification using the brightfield imaging setting on a Leica DMi8 microscope. After that, decrosslinking was performed immediately for H&E stained sections. Next, human whole transcriptome probe panels were then added to the tissue. After these probe pairs hybridized to their target genes and ligated to one another, the ligation products were released following RNase treatment and permeabilization. The ligated probes were then hybridized to the spatially barcoded oligonucleotides on the Capture Area. Spatial Transcriptomics libraries were generated from the probes and sequenced on the S4 flow cell of the Illumina NovaSeq 6000 system.

### FFPE spatial transcriptomics quantification and analysis

After cDNA library construction and sequencing, we use the short-read probe alignment algorithm for FFPE ‘count’ method in Space Ranger (v1.3.0) from the 10x Genomics to align probe read to the human reference genome (GRCh38). The resulting count matrix and associated H&E physiological images were then used by the R package Seurat (v.4.0.4)^122^ for subsequent analysis. The filtered gene-count matrices were normalized using SCTransform before being merged into one object for joint processing and analysis using the FindNeighbors, and FindClusters function in Seurat using standard processing parameters (30 PCs, original Louvain algorithm). The expression levels of selected genes were plotted using the function SpatialPlot and scaled to the same range.

### Cell lines

RCC4 line was purchased from a certified commercial vendor Sigma (https://www.sigmaaldrich.com/US/en/product/sigma/cb_03112702) and authenticated by STR profiling by Sigma. Caki-1 line (catalog number HTB-46, https://www.atcc.org/products/htb-46) and HEK293T (catalog number CRL-3216, https://www.atcc.org/products/crl-3216) were purchased from a certified commercial vendor ATCC and authenticated by STR profiling by ATCC. The SKRC-42 cells were from co-author Dr. James Hsieh’s lab https://www.cellosaurus.org/CVCL_6192 and were authenticated by sequencing analysis. No cell line used in this paper is listed in the database of commonly misidentified cell lines maintained by the International Cell Line Authentication Committee (ICLAC). All of the cell lines used here tested negative for mycoplasma contamination using InvivoGen MycoStrip (catalog: rep-mys-20).

### Lentiviral infection for delivery of CP, MXI1, and KLF9 into RCC4 and Caki-1 cells

The MISSION shRNA Bacterial Glycerol Stocks (catalog: SHCLNG, Sigma-Aldrich) for CP, MXI1, and KLF9 were used with two different constructs each as follows: CP_C1 with a target sequence being CCAGATAGAATTGGGAGACTA, CP_C2 with a target sequence being CCTACAGTATTTGATGAGAAT, MXI1_C1 with a target sequence being GCTCATTTCATGCTCTGCAAA, KLF9_C2 with a target sequence being AGTGATTCTGGGCCCTTTATG. All the aforementioned target sequences have been confirmed by Sanger sequencing (Genewiz). All vectors contain bacterial (ampicillin) and mammalian (puromycin) antibiotic resistance genes for the selection of inserts in either bacterial or mammalian cell lines. For the scrambled shRNA, MISSION® pLKO.1-puro Non-Mammalian shRNA Control Plasmid DNA (catalog: SHC002, Sigma-Aldrich), 500 ng/μL in TE buffer; DNA (10 μg of plasmid DNA), with mammalian (puromycin) antibiotic resistance genes. This was purchased as plasmid DNA and directly packaged into lentiviruses using HEK293T cells.

For the MISSION shRNA Bacterial Glycerol Stock, aliquots were streaked in an agar plate and grown overnight in a humidified incubator at 37 °C, after which a single colony was selected and amplified in agar broth overnight in an incubator shaker, and then purified using Qiagen Mini-Prep Kit for sequencing and then Qiagen Midi-Prep Kit for DNA purification according to the manufacturer’s protocol. HEK293T cells cultured in a complete culturing medium including 500 mL GibcoTM DMEM, high glucose, GlutaMAXTM Supplement, pyruvate with 10% GibcoTM FBS were used with Lipofectamine™ 3000 Transfection Reagent (catalog: L3000015 according to the manufacturer’s protocol in order to package the plasmid DNA into lentiviral particles. The transfection was done in T75 flasks of HEK293T cells at 95–99% confluency using 4.3 μg of the pLenti expression vector using ViraPower Lentiviral Packaging Mix (catalog: K497500) and Lentivirus packaging medium (500 mL GibcoTM Opti-MEMTM I Reduced Serum Medium, GlutaMAXTM Supplement with 1 mM GibcoTM Sodium Pyruvate and 5% Gibco FBS). Each MISSION Control Vector was provided as 10 mg of purified plasmid DNA at a concentration of ~500 ng/mL in 10 mM Tris-HCl, pH 8.0, containing 1 mM EDTA, with product titer (IU/ml) for the shRNA purchased: 1 × 10^6^ and 1 × 10^7^ TU/mL. The multiplicity of infection (MOI) used for both RCC4 and Caki-1 cell lines was ≈5 MOI. Puromycin was at 2 μg/ml for Caki-1 and 4 μg/ml for RCC4 cell line based on the Puromycin killing curve performed. Infection was performed using Polybrene (Catalog: TR-1003-G, Sigma-Aldrich) at a concentration of 8 μg/mL. The RCC4 and Caki-1 cell lines were maintained in Dulbecco’s modified Eagle medium/Nutrient Mixture F-12 (DMEM/F-12) culture medium (Gibco – 11320033) supplemented with 10% FBS (Sigma-Aldrich, F-9665) and 1% Pen Strep (Gibco, 10,000 U/mL−15140122). Puromycin selection was started 3 days post-infection and maintained throughout the culture maintenance. Cells were cultured in T75 flasks for western blot and T25 flasks for bulk RNA extractions.

### Western blotting

Cultured cells were washed with 1× PBS and lysed using 1x RIPA buffer (#9806, CST) and then centrifuged at 17,000 × *g* for 15 min. Supernatants were quantified using Bio-rad DC protein assay. Equal amounts of proteins were loaded and separated using 10% polyacrylamide gel. Proteins were transferred onto the activated PVDF membrane (Immobilon-FL Merck Millipore) and later blocked using Odyssey blocking buffer. Primary antibodies BTEB1 (A-5) or KLF9 (#sc-376422; Monoclonal (A-5); Santa Cruz) at 1:250, CP (#A80–124A; Polyclonal; Bethyl lab) at 1:1000, MXI1 (#12360-1-AP; Polyclonal; Proteintech) at 1:50, *β*-Tubulin (9F3 #2128S; Monoclonal; Cell Signaling Technology) at 1:1000, BAP1 (#sc-28383; Monoclonal (C-4); Santa Cruz) at 1:500, *β*-Actin (#3700S; Monoclonal (8H10D10); Cell Signaling Technology) at 1:5000 were incubated O/N at 4 °C and next day were incubated with Licor IR 680 #925-32214 (Donkey anti-Goat), Licor IR 680 #926-68072 (Donkey anti-Mouse), IR800 #926-32213 (Donkey anti-Rabbit) fluorescent antibodies and HRP-conjugated #715-035-150 (Donkey anti-Mouse) at 1:10,000 dilution. The blot was developed using the Bio-rad Chemidoc MP imaging system. Source data is provided as a Source data file.

### Bulk copy number calling

Copy number variation was detected using BIC-seq2^123^, a read depth-based CNV calling algorithm for WGS tumor data. BICseq2-norm (v.0.2.4) is for normalizing potential biases in the sequencing data. BICseq2-seg (v.0.7.2) is for detecting CNVs based on the normalized data given by BICseq2-norm. Briefly, BIC-seq2 divides genomic regions into disjoint bins and counts uniquely aligned reads for each bin. It then combines neighboring bins into genomic segments with similar copy numbers iteratively based on Bayesian information criteria (BIC). We used paired-sample CNV calling that takes a pair of samples as inputs and detects genomic regions with different copy numbers between the two samples. We used a bin size of 100 bp and a lambda of 3 (smoothing parameter for CNV segmentation). A segment was called copy gain if log_2_(copy ratio) was larger than 0.2 or copy loss if log_2_(copy ratio) was smaller than −0.2, respectively. To further summarize the arm-level copy number change, we used a weighted sum approach^124^, in which the segment-level log_2_(copy ratio) for all the segments located in the given arm were added up with the length of each segment being weighted.

### Sequencing read alignments and quality control (QC) of snRNA-seq data

After single-nuclei prep and sequencing, Cell Ranger (v3.1.0) from 10x Genomics (with Count functionality) was used for aligning reads to the human genome reference (GRCh38) with the addition of pre-mRNA reference (v3.0.0). The reference file was downloaded from the 10x Genomics website (https://support.10xgenomics.com/single-cell-gene-expression/software/downloads/latest). The pre-mRNA reference was added using the following code: awk ‘BEGIN{FS=“\t”; OFS=“\t”} $3 == “transcript”{$3=“exon”; print}’ refdata-cellranger-GRCh38-3.0.0/genes/genes.gtf > GRCh38-3.0.0.premrna.gtf; cellranger mkref --genome=GRCh38-3.0.0.premrna --fasta=refdata-cellranger-GRCh38-3.0.0/fasta/genome.fa --genes=GRCh38-3.0.0.premrna.gtf --nthreads 50. The parameters used with Count functionality include --chemistry=threeprime --expect-cells = 6000 --jobmode=local --localcores=20 --localmem = 350. The resulting gene-by-cell UMI count matrix was used by the R package Seurat (v.3.1.0)^122^ for all subsequent processing.

Quality filters were applied to the data to remove barcodes that fell into any of the following categories: too few genes expressed (possible debris), too many associated UMIs (possibly more than one cell), and too high mitochondrial gene expression (possible dead cell). The cut-offs for these filters were based on recommendations by Seurat package documentation and manually adjusted to keep the number of cells after filtering under 6500 (detailed filtering parameters see Supplementary Data 2). Finally, doublets were filtered out using Scrublet (v.0.2.1). Scrublet was run on each sample separately with the following parameter settings: expected_doublet_rate = 0.06, min_counts = 2, min_cells = 3, min_gene_variability_pctl = 85, n_prin_comps=30. The doublet score threshold was adjusted manually, which can separate the two peaks of a bimodal simulated doublet score histogram (see detailed thresholds used for each sample in Supplementary Data 2).

### Normalization, feature selection, and dimensional reduction of snRNA-seq data

The filtered gene-count matrix was normalized for sequencing depth by dividing by the total gene counts in each cell. The value was then log-transformed using the Seurat NormalizeData function (default parameters). We calculated a subset of features (genes) that showed high cell-to-cell variation for downstream analysis. For the processing of individual samples, the Seurat function FindVariableFeatures was used (with default parameters) to identify the top 2000 most variable features, which were then scaled using the Seurat function ScaleData (with the default parameters) to have respective mean expression and variance of 0 and 1 across cells. For the merging of datasets across all samples, the top 3000 most variable features were identified. Here, the features parameter for the ScaleData function was specified as all genes in the count matrix, whereby the downstream Principle Component Analysis (PCA) will take all features (with available scaled data) as inputs. To merge snRNA data from the same patient, we applied the Seurat function SCTransform with the parameter vars.to.regress specified as nCount_RNA and percent.mito. The scaled data were then used directly as input for PCA using the Seurat function RunPCA (with the default parameters). The first 30 Principal Components (PCs) were used for downstream analysis. We also used the RunUMAP function (with default parameters) and the first 30 PCs to perform the Uniform Manifold Approximation and Projection (UMAP), a standard dimensional reduction step, to visualize the snRNA data. For the processing of tumor cells only in individual samples and immune cells (lymphoid and myeloid lineage immune cells separately) of all samples, the same functions (used ScaleData instead of SCTransform) and the same aforementioned parameters were used.

### Clustering snRNA-seq data

Cells were clustered using a graph-based clustering (default of Seurat) approach. First, we utilized the Seurat function FindNeighbors to embed cells in a K-nearest neighbor (KNN) graph structure, based on the Euclidean distance in PCA space, with edges drawn between cells having similar expression patterns. We used the previously-defined first 30 PCs as inputs to the function, while other parameters were left as defaults. To cluster cells, we then applied modularity optimization techniques (using the default Louvain algorithm from the Seurat function FindClusters) to iteratively group cells together to optimize the standard modularity function. We set the resolution parameter at 0.5, while other parameters were left as defaults. For defining tumor clusters with substantial transcriptional differences, tumor-cell clusters initially assigned by Seurat (https://github.com/ding-lab/ccRCC_snRNA_analysis/blob/master/recluster/recluster_tumorcells/recluster_tumor_cells_in_selected_samples_rm_doublets_katmai.R) were visualized in UMAPs and manually inspected. Tumor-cell clusters without clear separation, suggesting a lack of transcriptional differences, were grouped into one cluster.

### Merging of snRNA-seq data across samples

We used the Seurat function merge to combine the Seurat objects from multiple samples after quality control. Details for merging, normalization, feature selection, dimension reduction, and clustering of all snRNA-seq datasets can be found at https://github.com/ding-lab/ccRCC_snRNA_analysis/blob/master/integration/seuratintegrate_34_ccRCC_samples/reciprocalPCA_integrate_34_ccRCC_samples.R, and details for the merging and downstream analysis for multiple samples from the same patient can be found at merge_same_patient_segments/merge_same_patient_segments_C3L-00088.R, both being at our code archive https://github.com/ding-lab/ccRCC_snRNA_analysis/blob/master/integration/.

### Cell-type annotation of snRNA-seq data

We curated from the literature a list of well-known markers, including *CA9* for tumor cells (a downstream target of HIF and commonly upregulated in ccRCC cells, but not in normal kidney cells) and *LRP2* for proximal tubule cells (Supplementary Data 2). Using the merged snRNA data, we filtered the marker genes down to those that were expressed in at least 10% of at least one cluster. We then labeled each cluster with cell type names by examining the expression values and the percentages expressed of all the filtered marker genes across all clusters (using the Dotplot function of the Seurat package). Finally, we also corrected the cell type labels in individual samples based on marker gene expression, mutation, and CNV mapping evidence.

### Tumor cell-associated marker discovery

Tumor-specific marker discovery was done in Seurat by comparing gene expression between tumor cells and non-tumor cells in patient samples. The pipeline consists of 4 steps: (1) compare expression levels across cell types strictly within samples to discern markers characteristic of tumor cells, identifying those that hold more generally across our 30 ccRCC samples (see detailed processing parameters at https://github.com/ding-lab/ccRCC_snRNA_analysis/blob/master/findmarkers/tumor_specific_markers/tumor_specific_markers_doparallel_V1.0.R), (2) narrow to those exclusive of proximal tubule (PT) cells and epithelial cell types (as they were scarce in tumor samples), (3) confirm their chromatin accessibility changes using snATAC-seq, and (4) validate in a larger cohort from bulk RNA and protein data, and further characterize using spatial transcriptomes.

Using this approach, we identified 324 ccRCC tumor-cell-specific markers from step 1. A gene is labeled tumor cell-specific if all of the following criteria are satisfied: (1) the average expression of the gene is higher in tumor cells compared with any other cell type, respectively, for at least one sample, and all the differences are statistically significant (log(Fold Change) >0; adjusted *P*-value < 0.05); (2) the average expression of the gene in tumor cells is higher compared with non-tumor cells (as a combined population) for 90% of the samples and that such diff was statistically significant in at least 75% of the samples; (3) the average expression of gene in tumor cells is higher compared to non-tumor cells in the normal tissue specifically. Finally, all *P*-values were adjusted by Bonferroni correction.

To find potential antigens, we further annotated tumor cell-specific genes by their subcellular location and tissue specificity. We used three databases to curate the subcellular location information: (1) Gene Ontology Term 0005886; (2) Mass Spectrometric-Derived Cell Surface Protein Atlas^125^ (CSPA); (3) The Human Protein Atlas (HPA) subcellular location data based on HPA version 19.3 and Ensembl version 92.38. Subsequently, we identified 120 candidate surface markers overexpressed in tumor cells compared to all the other cell types in a majority of individual samples (step 1), prioritizing 20 that were also overexpressed in ccRCC cells compared to normal proximal tubule cells and other epithelial cell types (step 2; Fig. 1c, adding the canonical ccRCC marker *CA9*), thereby bolstering specificity to ccRCC. 19 of these markers showed higher chromatin accessibility (gene activity, fold change >1) in tumor cells using snATAC-seq data, suggesting higher chromatin accessibility may contribute to their higher expression in tumor cells (step 3). 17 were further supported by the bulk RNA-seq and proteomics data, by comparing the tumors to the normal adjacent tissues of a larger cohort (step 4; Fig. 1c).

### Average expression of given genes by cell type and sample

For this analysis, we utilized the merged Seurat object with all the nuclei from all patients, and grouped nuclei by the combination of sample ID and cell type (set it as the identity of the nuclei using the Idents function). Then we used the AverageExpression function to calculate the average expression using the SCT assay and data slot of the Seurat object.

### Survival analysis

The R package survival (v. 3.2-13) was used to perform survival analysis. Kaplan–Meier curves of overall survival (function survfit) were used to compare prognoses among patients with high and low expression of tumor-cell-specific markers (https://github.com/ding-lab/ccRCC_snRNA_analysis/blob/master/clinical_association/survival/survfit_tumormarkers_by_tumorcell_snRNA_3groups_removemedium.R). The expression-high and expression-low groups were defined as those with expression level of the studied gene in the top and bottom 30% quantile respectively. The survival data were obtained from CPTAC clinical follow-up data as of Oct 2021.

### Differential expression analysis

To compare RCC cells (Caki-1) with *CP* knockdown (sh-CP-C1 and sh-CP-C2) vs. controls (sh-NT1 and sh-NT2) using bulk RNA-seq data, we used edgeR package (default parameters) to identify differentially expressed genes (only genes with counts > =2 in at least one sample were used).

To compare the tumor cells of each tumor sample vs. proximal tubule (PT) cells of four NATs using snRNA-seq data, we used the default test (Wilcoxon Rank-Sum test) of function FindMarkers (from the Seurat package) with the specified parameters: min.pct = 0.1, min.diff.pct = 0.1, log(fc.threshold) = 0, and only.pos = F. In addition, to correct for CNV, we performed a comparison of all tumor cells vs. normal PT cells using each pre-filtered gene and the corresponding CNV value calculated from bulk WGS data as latent variables. We removed from the final list of genes those that were insignificant after performing CNV correction. For comparing EMT vs. Epi-H tumor clusters and comparing the tumor cells of each of the *PBRM1*-mutant and *BAP1*-mutant tumors to the combined non-*BAP1*/*PBRM1*-mutated tumors, the following specified parameters were used: min.pct = 0.1, min.diff.pct = 0, log(fc.threshold) = 0, and only.pos = F.

For the filtering of differentially expressed genes (DEGs) consistently upregulated in tumor cells of individual tumors vs. combined PT cells, we require the DEGs to be significantly upregulated (p_val_adj <0.05, avg_logFC >0) in ≥50% of the comparisons. The filtering of DEGs consistently downregulated in tumor cells, and DEGs specific to *BAP1*- and *PBRM1*-mutant tumors individually and together was similar to the filtering strategy described above.

### Pairwise correlation of the gene expression of tumor cells and normal nephron epithelial cell types

First, we modified the tumor-cell-associated marker discovery pipeline to identify a set of markers specific to each of the 6 nephron epithelial cell types. Then we collected the top 100 cell-type-specific marker markers for each of the nephron epithelial cell types (see example at https://github.com/ding-lab/ccRCC_snRNA_analysis/blob/master/findmarkers/findmarkers_by_celltype/run_bycelltype_bysample.sh) and tumor cell. The average expression of the genes in the united gene list was used for the pairwise correlation of the cell groups.

### Calculating the pathway activity score using snRNA-seq data

To identify the top pathways that can best explain the variations among tumor subclusters within individual samples, we first identified differentially expressed genes (DEGs; positive only, otherwise default parameters) for each tumor subcluster (over 50 cells) for each tumor using the Seurat FindMarkers function (default parameters). Secondly, we ran over-representation tests for DEGs for each tumor subcluster using the “Hallmark” gene set from MSigDB database (to avoid gene redundancy) using the clusterProfiler package in R. Thirdly, we counted the frequency of a pathway over-represented in the subcluster-associated DEGs across tumors and focused on the pathways that enriched in at least one DEG set of tumor subcluster. Then we calculate the pathway scores for each tumor subcluster for each of the top pathways. For this step, we ran the AverageExpression function (Seurat package) to get the average expression of DEGs by tumor subclusters (SCT assay, data slot). For each DEG, its expression was scaled across all tumor clusters. And for each pathway, the pathway score is the average of the scaled expression of the pathway-associated DEGs for each tumor subcluster. For the pathway modules consisting of multiple pathways, tumor clusters with pathway scores in the upper 25% quantile for each member of the pathway module were considered enriched in the corresponding pathway module. For the mTOR pathway module, we require the tumor clusters to be in the upper 10% quantile for the pathway score of the “HALLMARK_MTORC1_SIGNALING” gene set to be considered enriched in the mTOR pathway module. For the EMT pathway module, we require the tumor clusters to be in the upper 10% quantile for the pathway score of the “HALLMARK_EPITHELIAL_MESENCHYMAL_TRANSITION” gene set and those with epithelial scores lower than 20% quantile to be considered enriched in the EMT pathway module. For comparisons of the pathway scores across patient groups, we took the highest pathway score across tumor subclusters in the same patient to be tested and visualized in these figures. Wilcoxon test was used to compare pathway scores between high tumor stage (stage III/IV) and low stage (stage I/II).

### Calculating epithelial score and assigning epithelial group using snRNA-seq expression

For the epithelial score, we used the markers for the proximal tubule cells and epithelial cells listed in Supplementary Data 2 that were also downregulated in the EMT-enriched tumor clusters vs. other tumor clusters (FDR < 0.05). We obtained their average expression by tumor subclusters and PT clusters using the AverageExpression function (Seurat package, SCT assay, “data” slot). The expression for each marker was scaled across all tumor-cell and PT clusters. And for each pathway, the epithelial score is the average of the scaled expression of the markers for each tumor subcluster. Tumor clusters with epithelial scores higher than 70% quantile were assigned as Epi-H tumor clusters. Tumor clusters with epithelial scores lower than 70% quantile and higher than 40% were assigned as Epi-M tumor clusters. Tumor clusters that were in neither of the above two groups nor EMT-enriched tumor clusters were assigned as Epi-L tumor clusters.

### Calculating tumor-cell-intrinsic signature scores using bulk RNA-seq

As immune cells may be the main contributor of inflammatory response gene expression in the bulk RNA-seq, we developed an analysis strategy to evaluate tumor-cell-intrinsic inflammatory response signature. First, we identify tumor subclusters with the top and bottom 10% quantile inflammatory response score. Second, we compared these two groups of tumor subclusters and identified genes overexpressed in the tumor clusters with top inflammatory response scores. Third, we overlapped these differentially expressed genes with tumor-cell-specific markers identified in the “Tumor cell-associated marker discovery” section. Finally, we calculate the signature score for each tumor in the CPTAC cohort using bulk RNA-seq by taking the mean value of the samplewise-scaled gene expression (log_2_FPKM). We performed survival analysis by comparing patients with top and bottom 25% quantile tumor-cell-intrinsic inflammatory response scores. Similarly, we also conducted signature calculation and survival analysis for six other gene sets associated with higher tumor grade.

### Sequencing read alignments and quality control (QC) of snATAC-seq

The Cell Ranger ATAC tool (v.1.2.0, 10x Genomics) was used to process the raw snATAC-seq data (FASTQ). We utilized the cellranger-atac count pipeline to filter and map snATAC-reads and to identify transposase cut sites. The GRCh38 human reference was used for the read mapping. Next, MACS2^126^ (v2.2.7.1) was used to perform peak calling. All peaks were resized to 501 bp centered at the peak summit defined by MACS2. After this, we combined all peaks and removed the ones overlapping with the peaks with greater signal, to get the set of non-overlapping peaks, as described in Schep et al.^127^. The resulting set of sample peaks was used to calculate the peak-count matrix using FeatureMatrix function from the R package Signac (v.1.2.0; https://github.com/timoast/signac), which was also used for downstream analysis. QC-filtering of the snATAC-seq data was performed using functions from the Signac package. Filters that were applied for the cell calling include: 1000 <number of fragments in peaks <20,000, percentage of reads in peaks >15, ENCODE blacklist regions percentage <0.05 (https://www.encodeproject.org/annotations/ENCSR636HFF/), nucleosome banding pattern score <10, and enrichment-score for Tn5-integration events at transcriptional start sites >2.

### Normalization, feature selection, and dimension reduction of snATAC-seq data

The filtered peak-count matrix was normalized using term frequency-inverse document frequency (TF-IDF) normalization implemented in the Signac package (parameters: method=1, scale.factor = 10,000). This procedure normalizes across cells, accounting for differences in coverage across them and across peaks, giving higher values to the more rare peaks. All the peaks were used as features for the dimensional reduction. We used the RunSVD function from Signac package to perform singular value decomposition on the normalized TF-IDF matrix using all peaks, which is known as Latent Semantic Indexing (LSI) dimension reduction. The resulting 2:30 LSI components were used for non-linear dimension reduction using the RunUMAP function from the Seurat package with parameter reduction = ’lsi’.

### Clustering of snATAC-seq data

The nuclei were clustered using a graph-based clustering approach implemented in Seurat. First, we utilized the Seurat function FindNeighbors to construct a Shared Nearest Neighbor graph using the 2:30 LSI components and specifying reduction = ’lsi’. Next, we used the FindClusters function to iteratively group nuclei together while optimizing modularity using the SLM algorithm.

### Merging of snATAC-seq data across samples

Merging of snATAC-seq datasets was performed using functions from the Signac and Seurat packages. In order to get the set of peaks for merging, we first combined peaks from all samples, and then for overlapping peaks, we performed an iterative removal procedure, the same as was used for creating individual sample sets of peaks. The resulting list of peaks was quantified in each dataset and was used to create a peak-cell matrix so that the set of features was the same across all snATAC datasets. After that, the merge function from the Seurat package was used to merge snATAC datasets. Next, we performed TF-IDF normalization. The LSI dimensional reduction was performed using the RunSVD function. Non-linear dimension reduction was performed using the RunUMAP function with the first 2:50 LSI components.

### Cell type label transfer from snRNA-seq to snATAC-seq data

Cell type label transfer was performed using functions from Signac and Seurat. First, we quantified chromatin accessibility associated with each gene by summing the reads overlapping the gene body and its upstream region of 2 kb, thus creating the gene-by-cell matrix. Coordinates for the genes were used from the Ensembl database v.86 (EnsDb.Hsapiens.v86 package). Next, we performed log-normalization of the resulting matrices using the NormaliseData function. The integration of paired snATAC-seq and snRNA-seq datasets was performed using the FindTransferAnchors function with the Canonical Correlation Analysis (CCA) option for dimensional reduction. We then utilized the TransferData function to transfer cell type labels from the snRNA-seq dataset to the snATAC-seq dataset using the obtained set of anchors from the previous step.

### Peak annotation

Peaks were annotated using R package ChiPseeker (v1.22.1) and the R package TxDb.Hsapiens.UCSC.hg38.knownGene (v.3.16.0). The promoter region was specified (−1000,100) relative to the transcription start site.

### Annotating differentially accessible chromatin regions (DACRs) with cis-regulatory elements

The R package CICERO^128^ (v.1.10.0) was used to annotate DACRs with cis-regulatory elements. Peaks co-accessible with the promoter peaks (co-accessibility cutoff 0.25) were annotated as potential enhancer elements.

### Calculation of TF motif scores using snATAC-seq data

To evaluate TF binding accessibility profiles in the snATAC-seq data, we used chromVAR^127^ (v1.6.0), which calculates biased-corrected deviations (motif scores) corresponding to gain or loss of accessibility for each TF motif relative to the average cell profile. Motif position frequency matrices were obtained from the JASPAR2020 R package (v.0.99.10).

### Identifying differential TF binding accessibilities between cell groups for snATAC-seq data

To compare the differences in the binding accessibility profiles between cell groups, we used a two-sided Wilcoxon rank-sum test, applying FDR correction for the resulting *p*-values. For the cell-type-specific TF motifs, we compared cells from each group vs. all other cells. For the comparison of tumor cells vs. the proximal tubule (PT) cells, we compared tumor cells for each sample vs. the PT cells pooled from four NAT samples. For Fig. 3a, we highlighted the motifs that are differentially accessible in >=50% of samples with red dots (To gray dots) and chose to further highlight motifs (in text) that are significantly more accessible in all tumor samples, to prioritize a small number of top significant TFs on which we concentrated our further analyses.

### Identifying ccRCC-specific TF motifs using bulk ATAC-seq data

To identify ccRCC-specific TFs in bulk ATAC-seq, we used data from the Corces et al.^46^ paper to search for KIRC-cohort-specific peaks. For this, we performed two comparisons: between samples from KIRC-cohort and samples from all other cancer types, and between samples from KIRC-cohort vs samples from KIRP-cohort. For downstream analysis, we used only significant peaks with positive fold change found in both comparisons. To calculate motif enrichment, we used TFmotifView^129^ with the default parameters.

### Identifying differentially accessible chromatin regions using snATAC-seq data

To identify differentially accessible chromatin regions (DACRs) between tumor cells and normal PT cells, we performed a comparison for tumor cells from each tumor sample vs. PT cells pooled from four NAT samples using the FindMarkers function from the Seurat package, with logistic regression test and the fraction of fragments in peaks as a latent variable to reduce the effect of different sequencing depths across cells. Bonferroni correction was applied for *P*-value adjustment using all peaks from the dataset. We required the peak to be significant (FDR < 0.05) in at least 50% of comparisons, and the same fold-change direction in all comparisons. In addition, to correct CNV, we performed a comparison of all tumor cells vs normal PT cells using a fraction of fragments in peaks and CNV-value calculated from bulk WGS data as latent variables. We removed from the final list of peaks the ones that were insignificant after performing CNV correction.

Next, to identify DACRs specific to *BAP1*-mutant and *PBRM1*-mutant tumors, we used the sets of samples for each category described above. We performed comparisons for each *BAP1*/*PBRM1*-mutant tumor sample vs pooled tumor cells from non-mutant samples. DACRs specific to *BAP1*-mutant and *PBRM1*-mutant groups of samples were selected if they were significantly more accessible in ≥50% of the samples from the respective groups compared to the non-mutant samples (we also required that a DACR should have the same fold-change direction in all comparisons). We chose to add this filter because we have a small number of *BAP1*-mutated samples and it was clear we would lack the necessary statistical power to assess whether the resulting peaks/genes would be consistently higher in the *BAP1*-mutated group. To be consistent, we applied this filtering strategy for differentially expressed genes of both *BAP1*-mutated tumors and *PBRM1*-mutated tumors. Finally, we removed peaks that were insignificant after CNV correction.

To calculate DACRs between Epi-H and EMT tumor clusters, we performed a comparison between the cells pooled from the two groups, using FindMarkers with logistic regression test and the fraction of fragments in peaks as a latent variable. To adjust P-values, Bonferroni correction was applied.

### Mapping TF motif to DACRs of DEGs

Then we filtered out genes lacking DACRs overlapping their short promoter regions (−1000 to 100 relative to the TSS). Next, we searched for motifs of top cell-type-specific TFs in the DACRs of selected DEGs for cell types of interest. We then divided the TF-DEG interactions into two categories based on the coordinates, relative to TSS, of the motifs found in the DACRs overlapping a promoter: (1) promoter motif, if the motif was found in the short promoter region (−1000 to 100 from TSS) and (2) distant motif, if the motif was found outside the promoter region. We used the described procedure to study the mechanisms of transcriptional regulation in both normal PT and tumor cells. Mapping of the motifs to the DACRs was performed using the motifmatchr (v1.8.0) R package.

### Visualizing the coverage of snATAC-seq for individual cell types

For snATAC coverage plots, we used the CoveragePlot function from the Signac package. For tumor samples, we plotted coverage for tumor cells only, and for NAT samples we plotted coverage for normal PT cells only.

### Over-representation test for differentially expressed genes and differentially accessible chromatin regions

For over-representation tests other than tumor subcluster-associated DEGs, we used the “Hallmark” gene set and the canonical gene set (v.7.4) from MSigDB^130,131^ database, and the enricher function from the clusterProfiler R package^132,133^. For the over-representation test of the DEGs between tumor cells and PT cells, genes that are expressed in at least 10% of either cell group were used as background. For the over-representation test for the DEGS with differentially accessible peaks associated with *BAP1* and *PBRM1* mutations, the nearest genes associated with all detected peaks were used as background. BAP1-associated pathways were selected by selecting gene sets with *P*-value <0.05 (<50% overlap).

### Reporting summary

Further information on research design is available in the Nature Portfolio Reporting Summary linked to this article.

## Supplementary information

Supplementary Information

Description of Additional Supplementary Files

Supplementary Data 1

Supplementary Data 2

Supplementary Data 3

Supplementary Data 4

Supplementary Data 5

Reporting Summary

## Data Availability

The raw snRNA-seq and snATAC-seq data files generated in this study have been deposited at the NCI Genomic Data Commons (GDC) and Cancer Data Service (CDS) under dbGAP accession code phs001287.v16.p6. Raw spatial transcriptomics and associated imaging data generated for this study have been deposited at the Human Tumor Atlas Network (HTAN) Data Coordinating Center Data Portal (https://data.humantumoratlas.org/), specifically HTAN WUSTL Atlas, under dbGAP accession code phs002371.v2.p1. Access to the raw data mentioned above requires dbGAP authorization, so as to protect the privacy and intent of research participants and to restrict data access to scientific investigators pursuing research questions consistent with the informed consent agreements provided by individual research participants. The requested raw data will be available as soon as dbGAP access has been granted. Additional requests for processed data can be addressed to L.D. (lding@wustl.edu) and will be responded to within one month. The publicly available raw and processed bulk WES and RNA-seq data files (generated by Clark et al.^13^) can be accessed through the GDC data portal under the CPTAC project page (https://portal.gdc.cancer.gov/projects/CPTAC-3). The publicly available raw and processed proteomics data and clinical data (generated by Clark et al.^13^) are available via the NCI Proteomics Data Commons. The publicly available processed bulk mutation, tumor purity, and immune subtype data were downloaded from the Clark et al. CPTAC study^13^. The human genome reference file was downloaded from the 10x Genomics website (https://support.10xgenomics.com/single-cell-gene-expression/software/downloads/latest). The subcellular location information can be retrieved from three databases: (1) Gene Ontology Term 0005886; (2) Mass Spectrometric-Derived Cell Surface Protein Atlas^125^ (CSPA); (3) The Human Protein Atlas (HPA) subcellular location data based on HPA version 19.3 and Ensembl version 92.38. The “Hallmark” and curated gene sets were downloaded from MSigDB. The processed data for re-generating all major figures are available through our public GitHub repository at https://github.com/ding-lab/ccRCC_sn_publication/tree/main/plot_data. The remaining data are available within the Article, Supplementary Information, or Source data file. Source data are provided with this paper.

## Source data

Source Data

## Code availability

The analysis codes for re-generating all major figures are available through the public GitHub repository at https://github.com/ding-lab/ccRCC_sn_publication.
