# Author Correction: Epigenetic and transcriptomic characterization reveals progression markers and essential pathways in clear cell renal cell carcinoma

**DOI:** 10.1038/s41467-023-38561-y

**Published:** 2023-05-17

**Authors:** Yige Wu, Nadezhda V. Terekhanova, Wagma Caravan, Nataly Naser Al Deen, Preet Lal, Siqi Chen, Chia-Kuei Mo, Song Cao, Yize Li, Alla Karpova, Ruiyang Liu, Yanyan Zhao, Andrew Shinkle, Ilya Strunilin, Cody Weimholt, Kazuhito Sato, Lijun Yao, Mamatha Serasanambati, Xiaolu Yang, Matthew Wyczalkowski, Houxiang Zhu, Daniel Cui Zhou, Reyka G. Jayasinghe, Daniel Mendez, Michael C. Wendl, David Clark, Chelsea Newton, Yijun Ruan, Melissa A. Reimers, Russell K. Pachynski, Chris Kinsinger, Scott Jewell, Daniel W. Chan, Hui Zhang, Aadel A. Chaudhuri, Milan G. Chheda, Benjamin D. Humphreys, Mehdi Mesri, Henry Rodriguez, James J. Hsieh, Li Ding, Feng Chen

**Affiliations:** 1 Oncology Division, Department of Medicine, Washington University in St. Louis, St. Louis, MO 63110 USA; 2 McDonnell Genome Institute, Washington University in St. Louis, St. Louis, MO 63108 USA; 3 Department of Pathology and Immunology, Washington University in St. Louis, St. Louis, MO 63110 USA; 4 Department of Genetics, Washington University in St. Louis, St. Louis, MO 63110 USA; 5 Department of Mechanical Engineering and Materials Science, Washington University in St. Louis, St. Louis, MO 63130 USA; 6 Department of Pathology, Johns Hopkins University, Baltimore, MD 21231 USA; 7 Van Andel Institutes, Grand Rapids, MI 49503 USA; 8 The Jackson Laboratory for Genomic Medicine, 10 Discovery Drive, Farmington, CT 06032 USA; 9 Department of Neurology, Washington University School of Medicine, St. Louis, MO 63110 USA; 10 Office of Cancer Clinical Proteomics Research, National Cancer Institute, Bethesda, MD 20892 USA; 11 Department of Radiation Oncology, Washington University School of Medicine, St. Louis, MO 63110 USA; 12 Siteman Cancer Center, Washington University in St. Louis, St. Louis, MO 63110 USA

**Keywords:** Cancer genomics, Tumour heterogeneity, Tumour biomarkers, Renal cell carcinoma

Correction to: *Nature Communications* 10.1038/s41467-023-37211-7, published online 27 March 2023

In the Acknowledgements section of this article, the grant number relating to US National Cancer Institute’s Clinical Proteomic Tumor Analysis Consortium given for Li Ding was incorrectly given as U24CA210976 and should have been U24CA210972. The original article has been corrected.

