# Supplementary material for: Epigenetic and transcriptomic characterization reveals progression markers and essential pathways in clear cell renal cell carcinoma
Source: Nat Commun. 2023 Mar 27;14:1681. doi: 10.1038/s41467-023-37211-7 (PMC10042888; doi:10.1038/s41467-023-37211-7)
Supplement: Supplementary file 1 — Supplementary Information [file 41467_2023_37211_MOESM1_ESM.pdf]

Supplementary Information

Chromatin accessibility shapes tumor architecture and microenvironment in clear cell renal cell carcinoma

Supplementary Figures 1-7
Supplementary Notes 1-2

Supplementary Figure 1

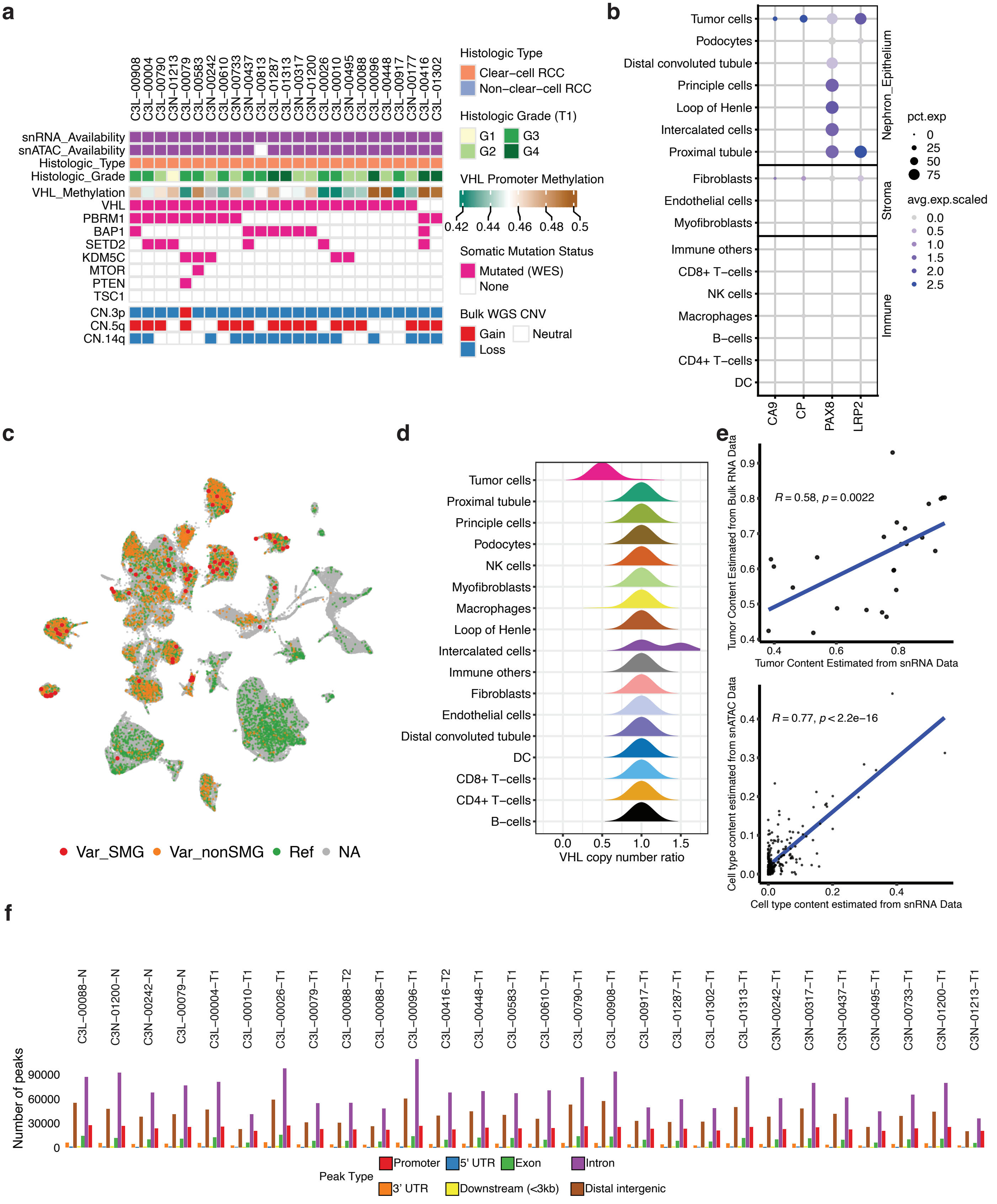

Supplementary Figure 1. Overview of snRNA-seq, snATAC-seq, and bulk omics data.
a, Genomic and clinical features for 25 ccRCC patients used in this study. **b**, Dot plot showing scaled snRNA-seq expression of ccRCC and proximal tubule cell markers across cell types. Overall, the identified tumor cells showed high expression levels of ccRCC tumor-cell markers. **c,** UMAP showing somatic mutations mapped to snRNA-seq data. Each dot represents a cell, colored by whether it's mapped with a mutated allele, whether the mutation is in significantly mutated genes (SMGs) or not, and whether it's mapped with a reference allele for the mutation. This UMAP shows the variant alleles, especially variants in genes known to be significantly mutated in ccRCC, are mostly mapped to tumor cell clusters shown in Fig. 1b, with reference alleles being well detected in the non-tumor cell clusters. **d,** Ridge plot showing the distribution of copy number ration for *VHL* gene in different cell types. The majority of the identified tumor cells show one copy loss in the *VHL* gene on chromosome 3p, a characteristic copy number alteration in ccRCCs, while the majority of the non-tumor cells showed no copy number alteration in *VHL*. **e**, Top: Scatter plot showing that tumor cell percentages estimated by the snRNA-seq (x-axis) were significantly correlated with the tumor purity estimates by ESTIMATE using bulk RNA data (y-axis) among 25 ccRCC tumors. Bottom: Scatter plot showing that cell type content estimated by snRNA-seq (x-axis) is significantly correlated with cell type content estimated by snATAC-seq (y-axis; n = 224; P-value = 7.2e-46). Statistical evaluation was performed using two-sided Student's t-tests. **f**, Bar plot showing the numbers of peaks, divided and colored by peak type, across the snATAC samples.

Supplementary Figure 2

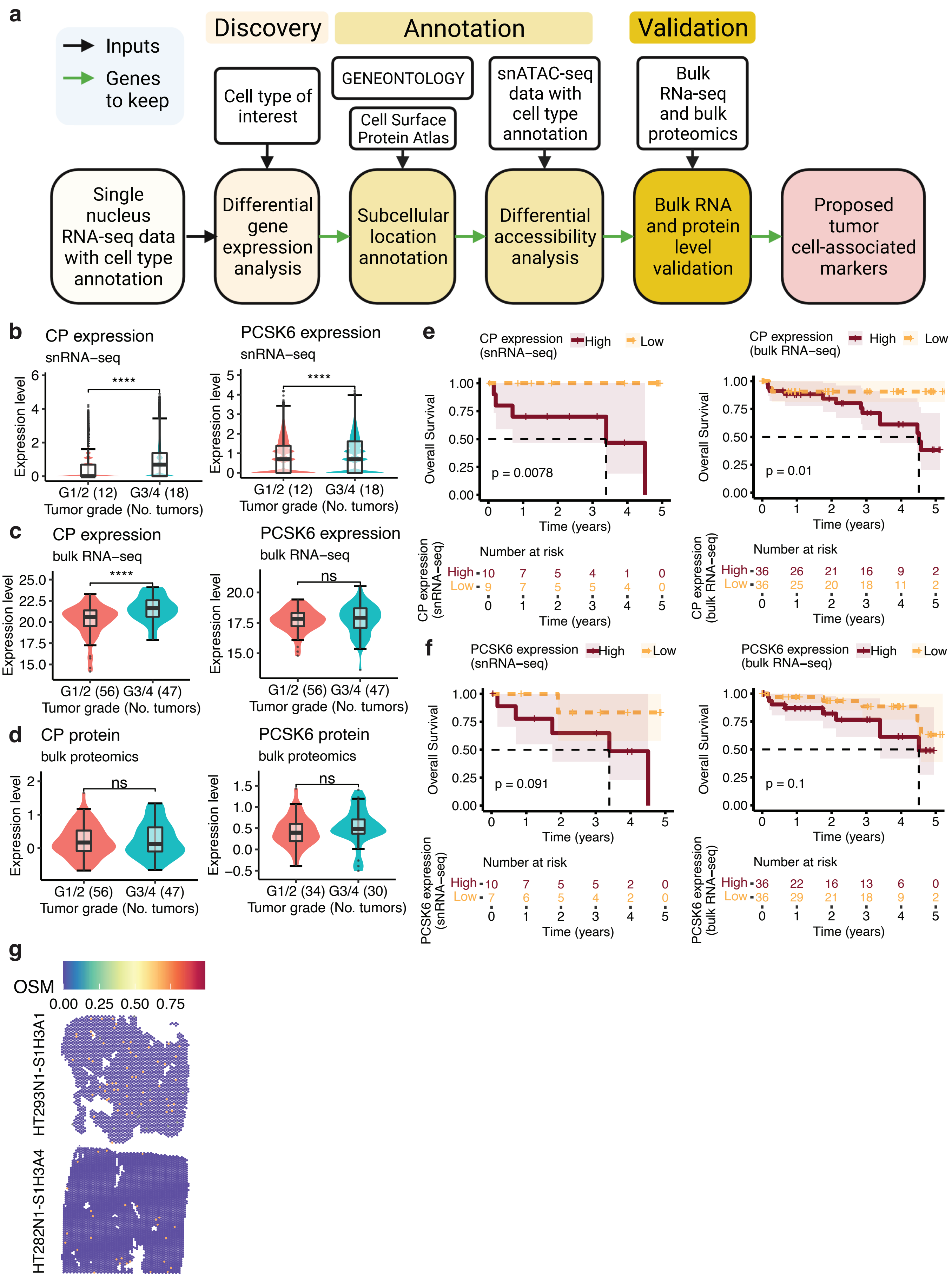

Supplementary Figure 2. *CP* expression is associated with higher tumor grades.
a, Schematic showing the tumor-cell discovery pipeline. Created with BioRender.com. **b**, Violin plots showing the tumor-cell expression of *CP* and *PCSK6* for low-grade (G1/2) and high-grade (G3/4) tumors (by cell). **c**, Violin plots showing the bulk RNA expression of the aforementioned genes for tumors with different grades. **d**, Violin plots showing the bulk protein abundance of the aforementioned genes for tumors with different grades. In **b**, **c**, and **d,** the box bounds the interquartile range divided by the median, with the whiskers extending to a maximum of 1.5 times the interquartile range beyond the box. Outliers are shown as dots. Student's T-tests; P-values are two-sided. Horizontal connecting segments indicate levels of statistically significant differences between grades (* = 0.05, ** = 0.01, *** = 0.001, **** = 0.0001, "ns" = not significant). **e**, Kaplan-Meier survival analysis showing overall survival after initial pathological diagnosis. Patients with a high tumor-cell expression of *CP* (n = 10, top 35% percentile) displayed a significantly lower chance of overall survival compared to patients with low tumor-cell expression of *CP* (n = 9, bottom 35% percentile) using snRNA-seq data (left plot). This result is validated by a similar analysis using bulk gene expression of *CP* in the larger CPTAC ccRCC discovery cohort (n = 66). **f**, Kaplan-Meier survival analysis showing overall survival after initial pathological diagnosis, separated by PCSK6 expression levels. In **e** and **f**, the ticks overlapping the lines represent censored data. The P-values were calculated by a two-sided log-rank test. **g,** Spatial transcriptomes of two ccRCC patient tumors showing *OSM* expression.

Supplementary Figure 3

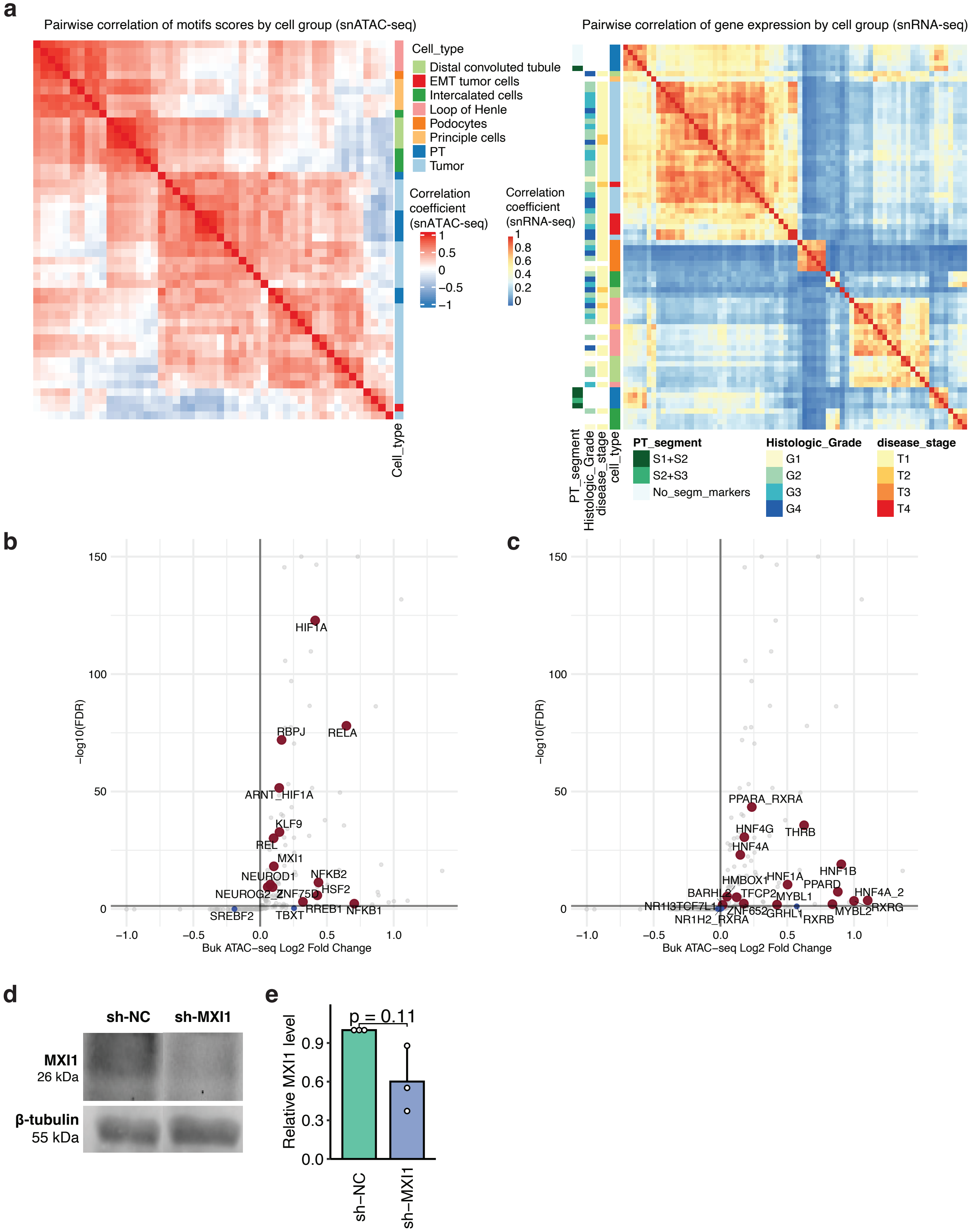

Supplementary Figure 3. Pairwise correlation between tumor cells and other normal nephron epithelial cell types, and bulk ATAC-seq validation of ccRCC-specific TF motifs.

a, Left: Heatmap showing the pairwise correlation between tumor cells and other normal nephron epithelial cell types using snATAC-based TF motif scores. Right: Heatmap showing the pairwise correlation between tumor cells and other normal nephron epithelial cell types using snRNA-seq-based gene expression. **b**, Volcano plot showing the snATAC-based ccRCC-specific TF motifs validated by the TCGA bulk ATAC-seq data (enriched in ccRCC compared to other cancer types, highlighted in red dots). Those that are not validated are in blue dots. **c**, Volcano plot showing the snATAC-based PT-specific TF motifs in the TCGA bulk ATAC-seq data. Red dots denote those that are significantly enriched in ccRCC compared to other cancer types. Those that are not enriched in ccRCC are in blue dots. **d,** Representative Western blot image showing MXI1 and beta-tubulin protein abundance in RCC4 MXI1 knockdown cells and scrambled control. **e,** Bar plot showing the Western blot densitometry values for MXI1 level in RCC4 *MXI1*-knockdown cells and scrambled control. The error bar represents the standard deviation of the mean from three independent experiments performed. Student's T-tests; P-values are two-sided.

Supplementary Figure 4

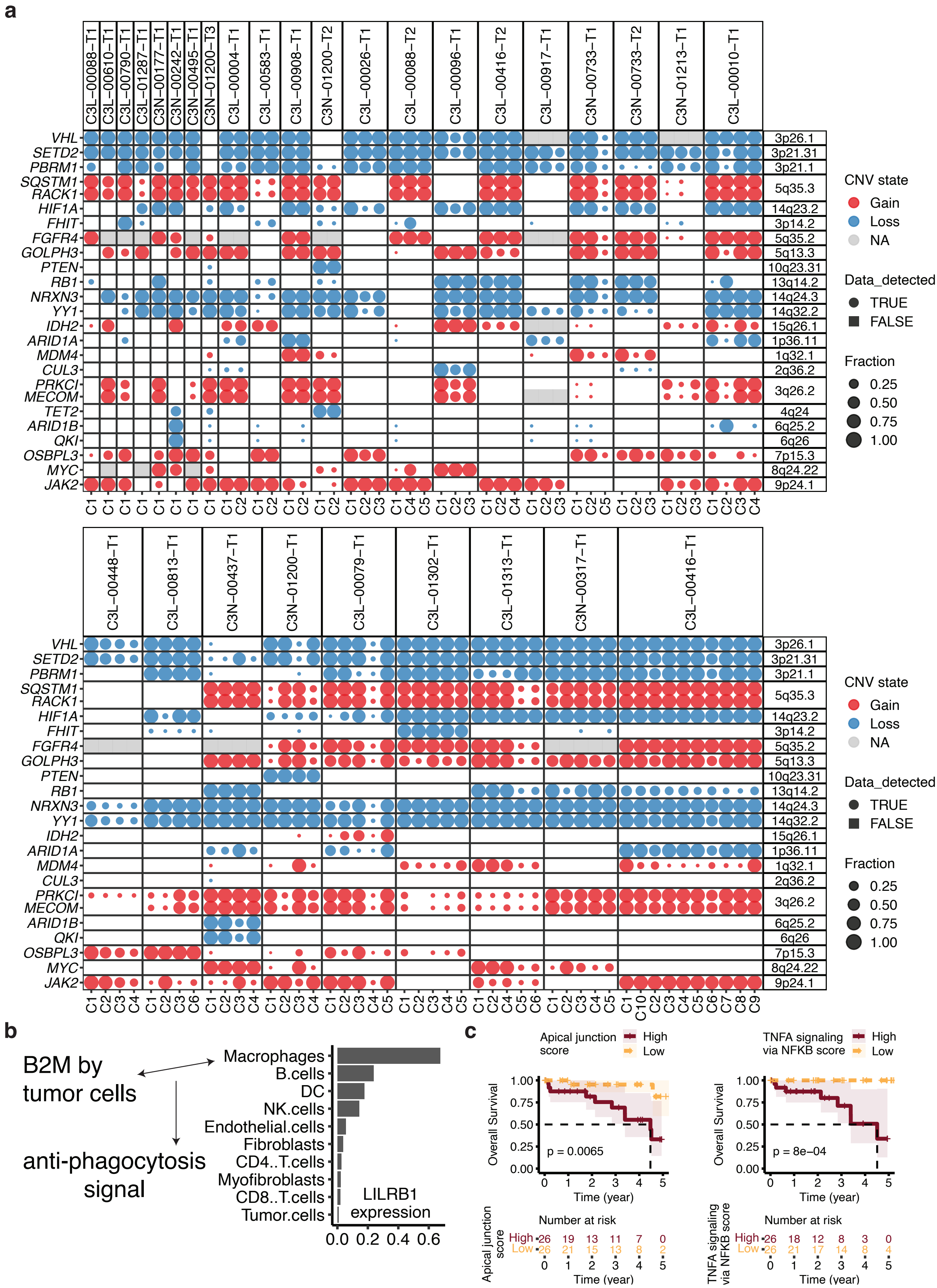

Supplementary Figure 4. Differential copy number status among tumor subclusters.
a, Bubble plot showing copy number status for tumor subclusters. **b**, Bar plot showing normalized expression of *LILRB1* across cell types and schematic showing the interaction between B2M and LILRB1. **c**, Kaplan-Meier survival analysis showing overall survival after initial pathological diagnosis. The P-values were calculated by a two-sided log-rank test. Patients with high tumor-cell-intrinsic apical junction score (n = 26, top 25% percentile) displayed a significantly lower chance of survival compared to patients with low apical junction score (n = 26, bottom 25% percentile) using bulk RNA-seq data. We identified a similar association between TNF-α signaling via NF-κB score and overall survival. The ticks overlapping the lines represent censored data.

Supplementary Figure 5

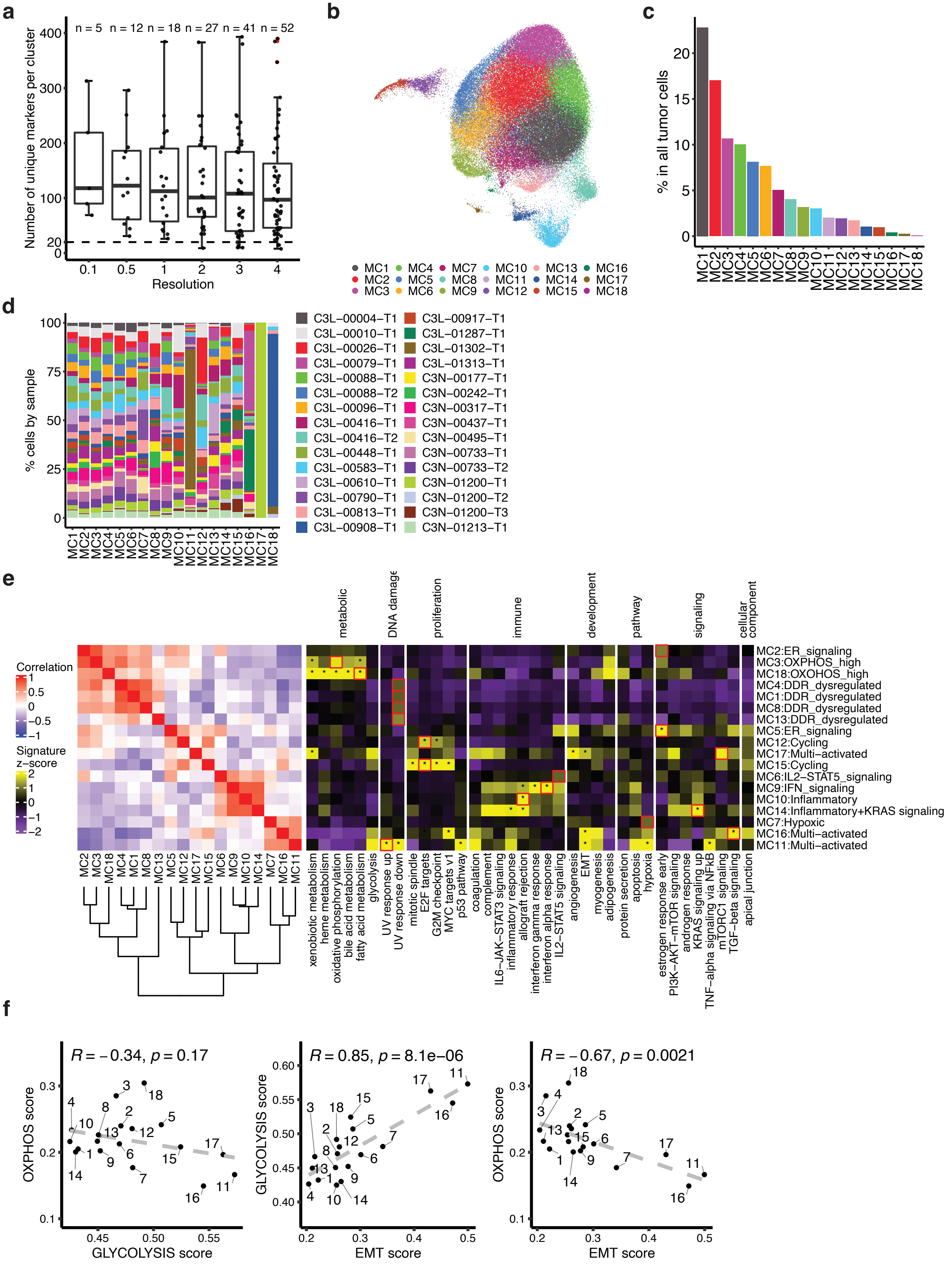

Supplementary Figure 5. Meta-cluster analysis.
a, Box plot showing the number of unique markers for meta-clusters generated by varying resolution parameters. The box bounds the interquartile range divided by the median, with the whiskers extending to a maximum of 1.5 times the interquartile range beyond the box. Outliers are shown as red dots. **b,** UMAP visualization of the integrated tumor-cell snRNA-seq data, colored by the meta-cluster number. **c,** Bar plot showing the % of cells in each cluster compared to all the tumor cells. **d,** Bar plot showing the % cells contributed by each tumor sample in each cluster, colored by sample. **e,** Left: Heatmap showing correlation coefficients of meta-clusters in terms of signature scores shown on the right. Right: Heatmap showing scaled signature scores of meta-clusters. The red rectangle border denotes the max z-score for a given meta-cluster across gene sets. * denotes the max z-score(s) for a given gene set across meta-clusters. **f,** Scatter plots showing: the glycolysis and OXPHOS signature scores (left panel), glycolysis and EMT signature scores (middle panel), and EMT and OXPHOS signature scores (right panel) across meta-clusters. Texts near the dots denote the meta-cluster number. Correlation coefficients and P-values were derived from two-sided Pearson correlation tests.

Supplementary Figure 6

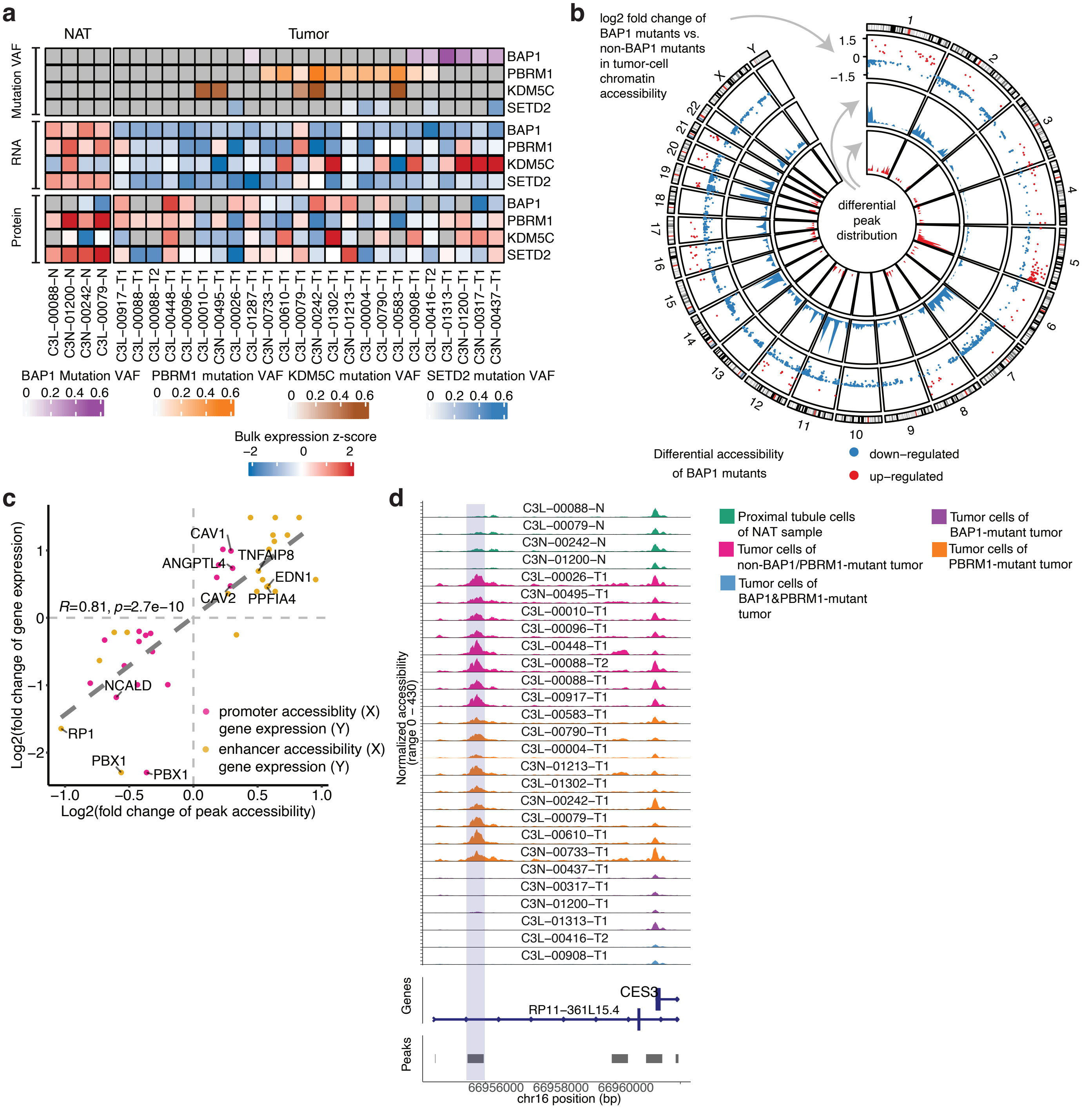

Supplementary Figure 6. Overview of the snATAC-seq samples and *BAP1/PBRM1*-associated chromatin accessibility changes.

a, Heatmap showing the mutation status, gene expression, and protein levels of the BAP1, PBRM1, KDM5C, and SETD2 among the snATAC-seq samples. **b**, Circos plot showing the distribution of ATAC-peaks associated with *BAP1* mutation. The outermost circles show the genomic location of individual ATAC-peaks associated with *BAP1* mutation, with red dots denoting more accessible peaks and blue dots denoting more closed peaks. The middle circle shows the density of more closed *BAP1*-associated peaks across the genome. The innermost circle shows the density of more *BAP1*-associated peaks across the genome. **c**, Scatter plot showing the positive correlation of chromatin accessibility and transcriptional changes associated with *PBRM1* mutation. The fold change (log2) of the snRNA-seq expression for each gene (mRNA) is plotted against the fold change (log2) in the relative snATAC-seq peaks (for all the genes/ peaks with significant fold change in over 50% of the comparisons for individual *PBRM1*-mutated tumor vs. tumors not mutated in either *BAP1* or *PBRM1*). Each dot represents a gene-peak pair. Dots are colored by whether the peak overlaps the gene promoter or is a potential enhancer (co-accessible with the promoter peak). **d**, Genomic region near *CES3*. The plots show the normalized accessibility signal by snATAC-seq around these regions in tumor cells of *BAP1*-mutant tumor (purple), tumor cells of *PBRM1*-mutant tumor (orange), tumor cells of non-*BAP1*/*PBRM1*-mutant tumors (pink), and proximal tubule cells (green) from NAT samples from representative tumor samples.

Supplementary Figure 7

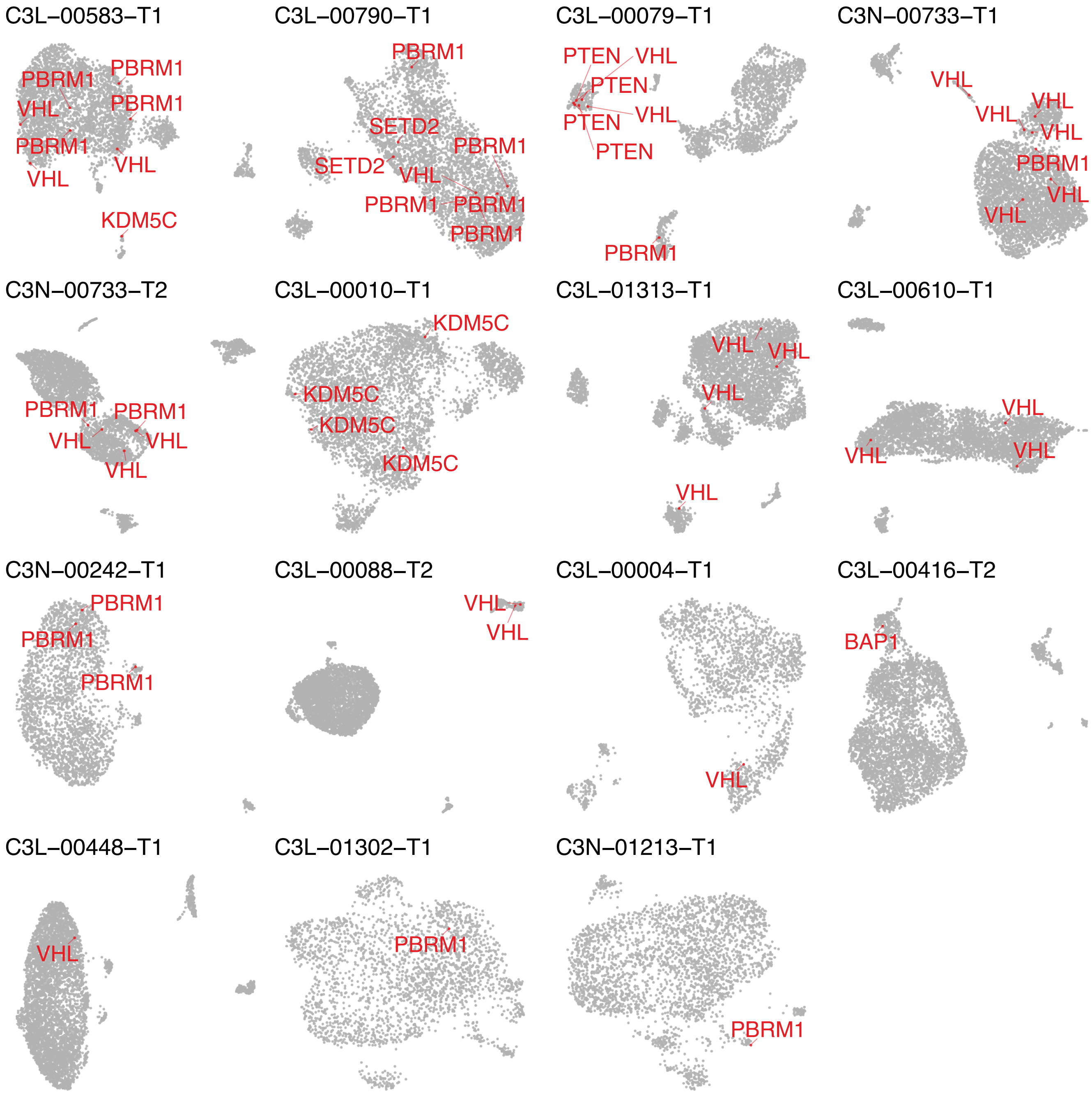

Supplementary Figure 7. Somatic mutations mapped to snRNA-seq data of tumor-cell clusters. UMAPs showing the tumor-cell clusters in individual tumor samples. Each dot denotes a cell and the red dot denotes a cell mapped with a somatic mutation in known significantly mutated genes detected in bulk WES data. Texts highlighted denote the genes in which the mutations were detected.

Supplementary Figure 8

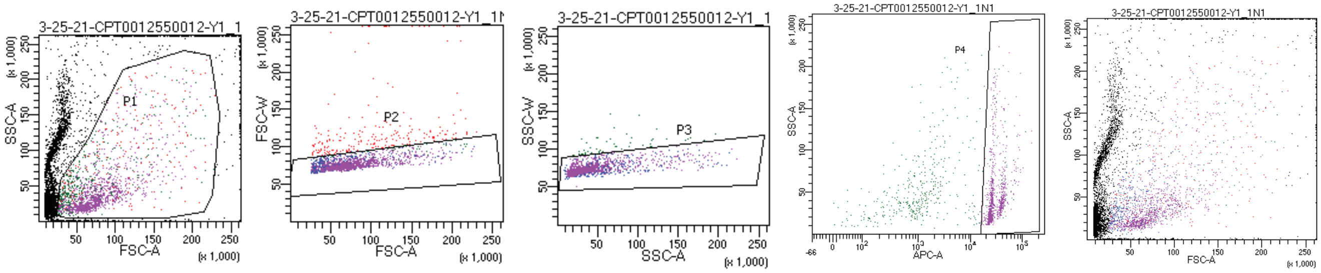

Tube: CPT0012550012-Y1_1N1

Population	#Events	%Parent	%Total
All Events	10,000	####	100.0
P1	1,718	17.2	17.2
P2	1,504	87.5	15.0
P3	1,453	96.6	14.5
P4	1,074	73.9	10.7

Supplementary Figure 8. Representative FACS plots portraying the gating strategy to isolate nuclei (P4 population).

Nuclei were stained with DRAQ5 and detected using the 640 nm laser for excitation and 670/30 dichroic filter for detection. Nuclei sequenced for ATAC were stained with 7-AAD and detected using the 488 nm or 561nm laser for excitation and 610/20 dichroic filter for detection. The P4 population was selected based on physical parameters forward scatter and side scatter (FSC-Area and SSC-Area), and doublet exclusion using the FSC-width and SSC-width).

Supplementary Figure 9

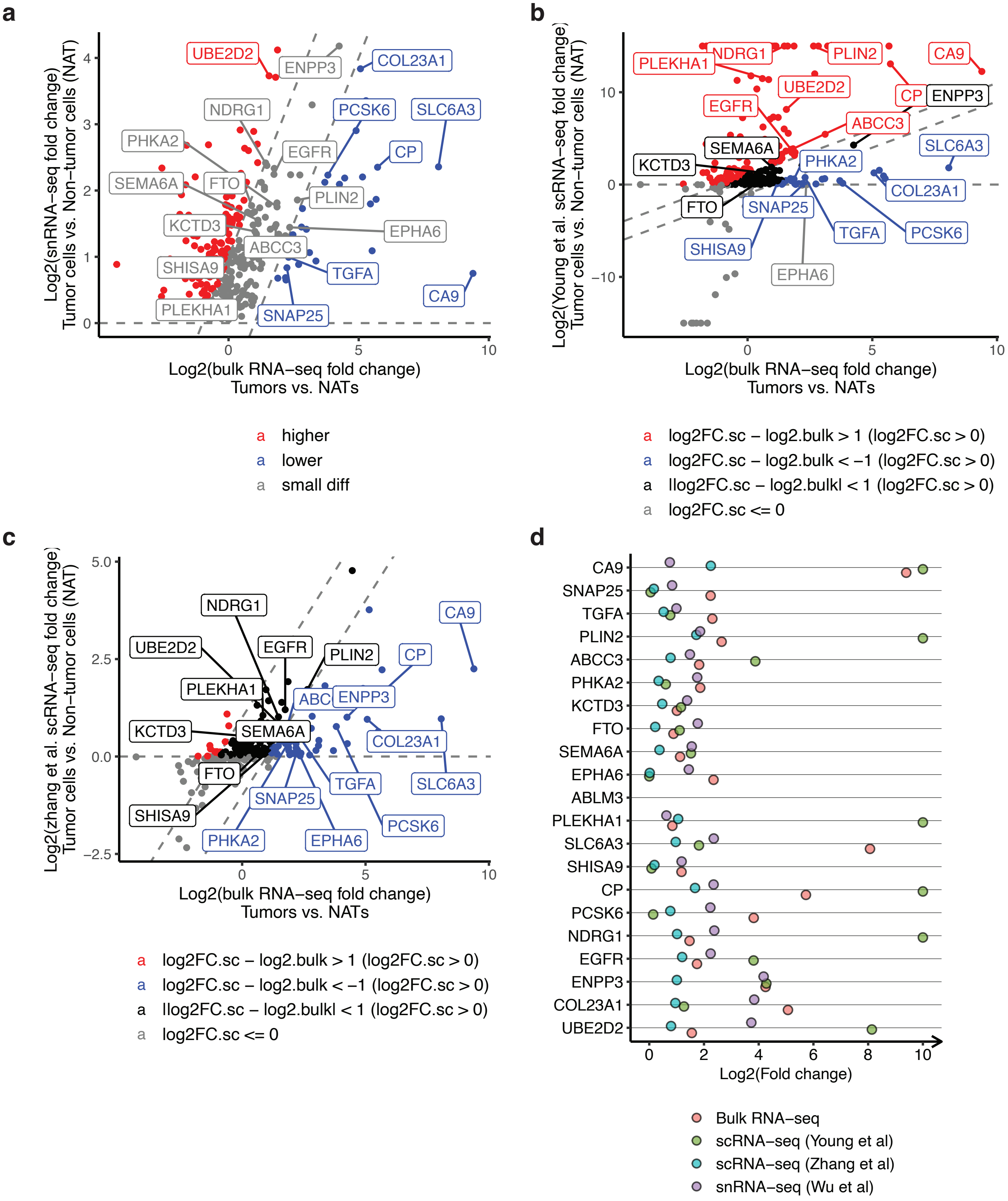

Supplementary Figure 9. Single-nucleus versus bulk RNA-seq data fold changes.
a, Scatter plot showing the Log2 fold changes for snRNA-based tumor-cell-markers calculated by bulk RNA-seq (x-axis) and snRNA-seq data (y-axis). Each dot represents one marker gene. Red and blue dots denote markers with higher and lower fold changes by snRNA-seq compared to bulk RNA-seq, respectively ($|\log_2$(fold change of sn data) - $\log_2$(fold change of bulk data)$| > 1$). Grey dots denote markers with similar fold changes by snRNA-seq and bulk RNA-seq ($|\log_2$(fold change of sn data) - $\log_2$(fold change of bulk data)$| <= 1$). Gene labels highlight the prioritized markers shown in the main figure. **b-c,** Scatter plot showing the Log2 fold changes for snRNA-based tumor-cell-markers calculated by bulk RNA-seq (x-axis) and scRNA-seq data (y-axis) published by Young et al. and Zhang et al. Each dot represents one marker gene. Black, red, and blue dots denote markers with higher expression in tumor cells than non-tumor cells ($\log_2$(fold change) > 0) using snRNA-seq data. Additionally, markers represented by red and blue dots showed higher and lower fold changes by scRNA-seq compared to bulk RNA-seq, respectively ($|\log_2$(fold change of sn data) - $\log_2$(fold change of bulk data)$| > 1$). Black dots denote markers with similar fold changes by scRNA-seq and bulk RNA-seq ($|\log_2$(fold change of sn data) - $\log_2$(fold change of bulk data)$| <= 1$). Grey dots denote markers with lower and same fold changes in tumor cells than non-tumor cells ($\log_2$(fold change) < 0) using snRNA-seq data. Gene labels highlight the prioritized markers shown in the main figure. **d,** Dot plot showing the log2 fold changes of expression of tumor-cell markers in ccRCC (capped at 10). Red dots denote fold changes by bulk RNA-seq data. Green and light blue dots denote fold changes by Young et al. and Zhang et al. scRNA-seq data. Purple dots denote the fold changes by the snRNA-seq data in this study.

Supplementary Note 1. Meta-cluster analysis. We performed a meta-cluster analysis to observe shared variations in the tumor-cell transcriptome across patients. We observed the tumor cells across patients aggregated into 18 meta-clusters (MCs). Specifically, we sub-clustered all tumor cells in our dataset and aligned sets of tumor cells derived from different samples to identify shared variations in cellular programs across the cohort. To determine the number of tumor-cell clusters, we ran the FindClusters function from the Seurat package, which identifies clusters of cells by a shared nearest neighbor (SNN) modularity optimization-based clustering algorithm. We tested six different clustering resolution parameters (ranging from 0.1 to 4) and counted the number of unique markers for each cluster. In order to obtain at least 20 unique markers for each cluster while producing the highest number of clusters, we chose the clustering resolution of 1 (Supplementary Fig. 5a). We obtained 18 meta-clusters (Supplementary Fig. 5b), and the percentages of tumor cells in each meta-cluster decreased from MC1 to MC18 (Supplementary Fig. 5c). Moreover, most of the clusters were derived from all samples except MC11, MC16, MC17, and MC18, which were more sample-specific (Supplementary Fig. 5d).

To identify the cellular programs underlying their transcriptional differences, we scored the tumor cells for the expression of the Hallmark gene signatures from MSigDB (Supplementary Fig. 5e) using VISION[1], the same tool used by the Bi et al. study[2]. These gene sets were selected based on significant local consistency of the signature scores ($C' > 0.1$ and $FDR < 0.05$). Several groups of meta-clusters showed high pairwise correlations and enriched signature scores in metabolism, immune signaling, proliferation, and DNA damage-related pathways. For example, MC6, MC9, MC10, and MC14 demonstrated high pairwise correlations and high activities in immune-related pathways, such as the interferon-gamma pathway and the inflammatory response pathway. MC12 and MC15 were also highly correlated and showed high signature scores in cell cycle-related pathways. In addition, MC3 and MC18 displayed high signature scores for oxidative phosphorylation (OXPHOS), fatty acid and bile acid metabolism, and adipogenesis. Moreover, MC1, MC4, MC8, and MC13 have the highest relative activities in down-regulated genes in response to ultraviolet (UV) radiation and scored relatively low in all the other pathways. We also observed four meta-clusters with activation across multiple types of gene sets, namely MC11, MC16, MC17, and MC18. All four meta-clusters seem to be more sample-specific (Supplementary Fig. 5e). Three out of the four meta-clusters showed the highest EMT scores, namely MC11, MC16, and MC17 (Supplementary Fig. 5f). They mainly were derived from 4 patients with stage III/IV disease, suggesting they may be precursors of the metastatic tumor cells.

Gene expression of various metabolic pathways is one of the differentiating features across meta-clusters. Several meta-clusters exhibit the most extreme activity in metabolic pathways. MC3 and MC18 (10.67% and 0.06% of all tumor cells, respectively) showed the highest OXPHOS scores and median glycolysis scores. In contrast, MC11, MC16, and MC17 (2.01%, 0.39%, and 0.24% of tumor cells, respectively) showed the highest glycolysis scores and lowest OXPHOS scores (Supplementary Fig. 5f). We observe a weak negative association between glycolysis and OXPHOS gene signatures. This association is expected, as cells under hypoxia conditions enhance glycolysis to compensate for weakened OXPHOS activity[3]. ccRCC cells were generally found to display activated glycolysis and inhibited mitochondrial OXPHOS[4,5]. Our results demonstrate the advantage of snRNA-seq in revealing tumor subpopulations with differential glycolysis and OXPHOS gene activities and may provide valuable information for studying glycolysis-targeting drugs in ccRCC[6]. Interestingly, we observed a significant positive correlation between glycolysis and EMT gene signature ($R = 0.85$, $P < 0.001$). In particular, MC11, MC16, and MC17 scored highest in glycolysis and EMT gene signatures. This result suggests that a subset of ccRCC primary tumor cells may up-regulate glycolysis activity to meet the increased energy demands of transitioning to a more mesenchymal state, a phenomenon that has been reported in other cancers[7].

Previously, Bi and colleagues[2] had found a tumor population (TP2) exhibiting both up-regulated glycolysis and OXPHOS gene signatures relative to tumor population TP1. However, in our analysis, we did not observe any meta-cluster with the highest glycolysis signature and also with the highest OXPHOS

signature. We believe there are several possible explanations for these inconsistent observations. Firstly, the samples used by BI et al. consist mostly of metastatic and stage III/IV ccRCC, while our samples consist only of primary ccRCCs, ranging from stage I to stage IV. Compared to the primary tumor site, ccRCC cells at the metastatic site may exhibit differential metabolic activity to meet the demands of different cellular conditions and tumor microenvironments[8]. Secondly, our analysis used a considerably larger set of tumors (n = 30) compared to the Bi et al. study (n = 8). So we may be able to capture rarer and more diverse tumor subpopulations that were not captured by BI et al. Thirdly, the gene signatures are relative within the tumor cells. Assuming we only captured three meta clusters, e.g., MC10, MC15, and MC16 (they are selected as all three showing high EMT signature and mainly derived from stage III/IV patients), we would have observed that both MC15 and MC16 showed higher glycolysis and OXPHOS gene signature compared to MC10, which is similar to the difference between TP2 and TP1 in terms of these two pathways reported by Bi et al.

Supplementary Note 2. Comparison with bulk RNA-seq data and published ccRCC single-cell data. Of the 21 prioritized tumor markers, 12 markers showed lower snRNA-based fold changes compared to bulk RNA-seq, which satisfy $\log_2$(fold change of bulk data) - $\log_2$(fold change of sn data) > 1. Three marker showed higher fold change, which satisfy $\log_2$(fold change of sn data) - $\log_2$(fold change of bulk data) > 1. And six markers showed similar fold changes, which satisfy |$\log_2$(fold change of sn data) - $\log_2$(fold change of bulk data)| <= 1.

To see if snRNA-based fold changes for tumor-cell markers are generally smaller compared to bulk RNA-seq, we looked through 324 tumor-cell markers discovered by snRNA-seq (that are also detected in bulk RNA-seq) and found that overall a majority of tumor markers (90%) showed higher or similar fold changes by snRNA-seq compared to bulk RNA-seq (Supplementary Fig. 9a). Of note, we derived the bulk RNA-seq fold changes by comparing expression in tumors (mostly tumor cells, stroma, and immune cells) vs. expression in NATs (mostly normal epithelial cells, stroma, and immune cells). To make the fold changes more comparable, for snRNA-seq data we compared the expression in tumor cells (in tumor tissue) vs. all cells in the NATs. 40.5% showed higher fold change (e.g. UBE2D2), 49.5% showed similar fold change (e.g. ENPP3), and 10.0% showed lower fold change by snRNA-seq compared to bulk RNA-seq (e.g. CA9). Genes that showed lower fold change by snRNA-seq compared to bulk RNA-seq may underlie the difference between the two technologies: snRNA-seq mostly measures nuclear transcripts, while bulk RNA-seq measures both cytoplasmic and nuclear transcripts.

We also analyzed scRNA-seq data for ccRCC samples published by Young et al.[55] and Zhang et al.[56]. Overall, 83.6% and 68.8% of the snRNA-based tumor-cell markers (n = 305; only used genes detected in all three studies) showed higher expression in tumor cells compared to non-tumor cells in the Young et al. and Zhang et al. scRNA-seq data, respectively, supporting most of these markers could help distinguish tumor cells from non-tumor cells. These include almost all of the prioritized snRNA-based markers (Supplementary Fig. 9b-c; except for EPHA6).

Assuming some tumor-cell markers may be more cell-enriched rather than nucleus-enriched, their fold change measured by snRNA-seq might be lower, but their fold change by scRNA-seq might be similar or higher compared to that measured by bulk RNA-seq. Of those markers that showed lower fold change in snRNA-seq compared to bulk RNA-seq, 46.7% (e.g. *CA9* and *CP*) showed higher or similar fold change in Young et al. scRNA-seq data compared to bulk RNA-seq (Supplementary Fig. 9b), suggesting transcripts for these genes potentially are more cell-enriched in the corresponding experimental condition. However, none of the snRNA-based markers with smaller fold change than bulk RNA-seq showed higher fold change calculated by the Zhang et al. scRNA-seq data compared to bulk RNA-seq (Supplementary Fig. 9c), suggesting the relative expression in tumor cells vs. non-tumor cells may vary by experimental condition. Different studies used different experimental protocols. For example, cells in the Young et al. study underwent FACS sorting and enrichment of immune cells by CD45 expression, while Zhang et al.

did not mention such procedures. There are still many genes that showed lower fold changes in both sn and scRNa-seq compared to bulk RNA-seq, such as *SLC6A3* and *COL23A1* (Supplementary Fig. 9d), which might underlie the difference between sn/scRNA-seq and bulk RNA-seq experimental processing. The different methodological protocols may result in different cell/nucleus states, which may affect post-transcriptional modification/stability and the measurement of the final transcript abundance.

References:

1. DeTomaso, D. *et al.* Functional interpretation of single cell similarity maps. *Nat. Commun.* **10**, 4376 (2019).
2. Bi, K. *et al.* Tumor and immune reprogramming during immunotherapy in advanced renal cell carcinoma. *Cancer Cell* **39**, 649-661.e5 (2021).
3. Zheng, J. Energy metabolism of cancer: Glycolysis versus oxidative phosphorylation (Review). *Oncol. Lett.* **4**, 1151–1157 (2012).
4. Nilsson, H. *et al.* Primary clear cell renal carcinoma cells display minimal mitochondrial respiratory capacity resulting in pronounced sensitivity to glycolytic inhibition by 3-Bromopyruvate. *Cell Death Dis.* **6**, e1585–e1585 (2015).
5. Wettersten, H. I., Aboud, O. A., Lara, P. N. & Weiss, R. H. Metabolic reprogramming in clear cell renal cell carcinoma. *Nat. Rev. Nephrol.* **13**, 410–419 (2017).
6. van der Mijn, J. C., Panka, D. J., Geissler, A. K., Verheul, H. M. & Mier, J. W. Novel drugs that target the metabolic reprogramming in renal cell cancer. *Cancer Metab.* **4**, 14 (2016).
7. Bhattacharya, D. & Scimè, A. Metabolic Regulation of Epithelial to Mesenchymal Transition: Implications for Endocrine Cancer. *Front. Endocrinol.* **10**, 773 (2019).
8. Bergers, G. & Fendt, S.-M. The metabolism of cancer cells during metastasis. *Nat. Rev. Cancer* **21**, 162–180 (2021).
