# Supplementary material for: Epigenetic and transcriptomic characterization reveals progression markers and essential pathways in clear cell renal cell carcinoma
Source: Nat Commun. 2023 Mar 27;14:1681. doi: 10.1038/s41467-023-37211-7 (PMC10042888; doi:10.1038/s41467-023-37211-7)
Supplement: Supplementary file 2 — Description of Additional Supplementary Files [file 41467_2023_37211_MOESM2_ESM.pdf]

## Description of Additional Supplementary Files

File Name: Supplementary Data 1
Description: Clinical information of the participants and specimen information.

File Name: Supplementary Data 2
Description: Marker genes used for cell-type annotation.

File Name: Supplementary Data 3
Description: Tumor-specific markers and differentially expressed genes and differentially accessible peaks and TF motifs.

File Name: Supplementary Data 4
Description: Selected pathways, epithelial, and EMT scores among tumor clusters.

File Name: Supplementary Data 5
Description: BAP1/PBRM1-associated differentially expressed genes and accessible peaks.
