# Supplementary material for: Epigenetic and transcriptomic characterization reveals progression markers and essential pathways in clear cell renal cell carcinoma
Source: Nat Commun. 2023 Mar 27;14:1681. doi: 10.1038/s41467-023-37211-7 (PMC10042888; doi:10.1038/s41467-023-37211-7)
Supplement: Supplementary file 8 — Reporting Summary [file 41467_2023_37211_MOESM8_ESM.pdf]

nature portfolio

Corresponding author(s): Li Ding

Last updated by author(s): Feb 17, 2023

Reporting Summary

Nature Portfolio wishes to improve the reproducibility of the work that we publish. This form provides structure for consistency and transparency in reporting. For further information on Nature Portfolio policies, see our Editorial Policies and the Editorial Policy Checklist.

Statistics

For all statistical analyses, confirm that the following items are present in the figure legend, table legend, main text, or Methods section.

n/a	Confirmed	
☐	☒	The exact sample size (*n*) for each experimental group/condition, given as a discrete number and unit of measurement
☐	☒	A statement on whether measurements were taken from distinct samples or whether the same sample was measured repeatedly
☐	☒	The statistical test(s) used AND whether they are one- or two-sided<br>*Only common tests should be described solely by name; describe more complex techniques in the Methods section.*
☐	☒	A description of all covariates tested
☐	☒	A description of any assumptions or corrections, such as tests of normality and adjustment for multiple comparisons
☐	☒	A full description of the statistical parameters including central tendency (e.g. means) or other basic estimates (e.g. regression coefficient) AND variation (e.g. standard deviation) or associated estimates of uncertainty (e.g. confidence intervals)
☐	☒	For null hypothesis testing, the test statistic (e.g. *F*, *t*, *r*) with confidence intervals, effect sizes, degrees of freedom and *P* value noted<br>*Give P values as exact values whenever suitable.*
☒	☐	For Bayesian analysis, information on the choice of priors and Markov chain Monte Carlo settings
☐	☒	For hierarchical and complex designs, identification of the appropriate level for tests and full reporting of outcomes
☐	☒	Estimates of effect sizes (e.g. Cohen's *d*, Pearson's *r*), indicating how they were calculated

Our web collection on statistics for biologists contains articles on many of the points above.

Software and code

Policy information about availability of computer code

Data collection	No commercial, open source and custom code were used to collect the data in this study.
Data analysis	The processed data and analysis codes for re-generating almost all major figures are available through the public GitHub repository at https://github.com/ding-lab/ccRCC_sn_publication.<br><br>The processed data and analysis codes for re-generating almost all major figures are available through the public GitHub repository at https://github.com/ding-lab/ccRCC_sn_publication.<br><br>The following open-source software/code was also used to analyze the data in this study:<br>- Scrublet v.0.2.1 was used to infer potential doublets in the snRNA-seq data.<br>- BICseq2-norm (v.0.2.4) is for normalizing potential biases in the sequencing data. BICseq2-seg (v.0.7.2) is for detecting CNVs based on the normalized data given by BICseq2-norm.<br>- Cell Ranger (v3.1.0) from 10X Genomics (with Count functionality) was used for aligning reads to the human genome (GRCh38) with the addition of pre-mRNA reference (v3.0.0). The resulting gene-by-cell UMI count matrix was used by the R package Seurat (v.3.1.0) for all subsequent processing.<br>- R version 4.1.2<br>- The R package survival (v. 3.2-13) was used to perform survival analysis. Kaplan-Meier curves of overall survival (function survfit) were used to compare prognoses among patients with high and low expression of tumor-cell-specific markers<br>- The Cell Ranger ATAC tool (v.1.2.0, 10X Genomics) was used to process the raw snATAC-seq data (FASTQ)<br>- MACS2 v2.2.7.1 was used to perform peak calling<br>- Signac package v.1.2.0 was used to calculate the peak-count matrix using FeatureMatrix function

- Peaks were annotated using R package ChiPseeker (v1.22.1)
- The R package CICERO (v.1.10.0) was used to annotate DACRs with cis-regulatory elements.
- To evaluate TF binding accessibility profiles in the snATAC-seq data, we used chromVAR (v1.6.0), which calculates biased-corrected deviations (motif scores) corresponding to gain or loss of accessibility for each TF motif relative to the average cell profile.
- To calculate motif enrichment, we used TFmotifView129 with the default parameters.
- Mapping of the motifs to the DACRs was performed using the motifmatchr (v1.8.0) R package.

For manuscripts utilizing custom algorithms or software that are central to the research but not yet described in published literature, software must be made available to editors and reviewers. We strongly encourage code deposition in a community repository (e.g. GitHub). See the Nature Portfolio guidelines for submitting code & software for further information.

Data

Policy information about availability of data

All manuscripts must include a data availability statement. This statement should provide the following information, where applicable:

- Accession codes, unique identifiers, or web links for publicly available datasets
- A description of any restrictions on data availability
- For clinical datasets or third party data, please ensure that the statement adheres to our policy

Data availability
The raw snRNA-seq and snATAC-seq data files generated in this study have been deposited at the NCI Genomic Data Commons (GDC) and Cancer Data Service (CDS) under dbGAP accession code phs001287.v15.p6 (https://www.ncbi.nlm.nih.gov/projects/gap/cgi-bin/study.cgi?study_id=phs001287.v15.p6). Raw spatial transcriptomics and associated imaging data generated for this study have been deposited at the Human Tumor Atlas Network (HTAN) Data Coordinating Center Data Portal (https://data.humantumoratlas.org/), specifically HTAN WUSTL Atlas, under dbGAP accession code phs002371.v2.p1 (https://www.ncbi.nlm.nih.gov/projects/gap/cgi-bin/study.cgi?study_id=phs002371.v2.p1). Access to the raw data mentioned above requires dbGAP authorization, so as to protect the privacy and intent of research participants and to restrict data access to scientific investigators pursuing research questions consistent with the informed consent agreements provided by individual research participants. The requested raw data will be available as soon as dbGAP access has been granted. Additional requests for processed data can be addressed to L.D. (lding@wustl.edu) and will be responded to within one month. The publicly available raw and processed bulk WES and RNA-seq data files (generated by Clark et al.13) can be accessed through the GDC data portal under the CPTAC project page (https://portal.gdc.cancer.gov/projects/CPTAC-3). The publicly available raw and processed proteomics data and clinical data (generated by Clark et al.13) are available via the NCI Proteomics Data Commons (https://pdc.cancer.gov/pdc/browse/filters/pdc_study_id:PDC000127%7CPDC000128%7CPDC000129%7CPDC000130). The publicly available processed bulk mutation, tumor purity, and immune subtype data were downloaded from the Clark et al. CPTAC study13.

The human genome reference file was downloaded from the 10X Genomics website (https://support.10xgenomics.com/single-cell-gene-expression/software/downloads/latest). The subcellular location information can be retrieved from three databases: 1) Gene Ontology Term 0005886; 2) Mass Spectrometric-Derived Cell Surface Protein Atlas125 (CSPA); 3) The Human Protein Atlas (HPA) subcellular location data based on HPA version 19.3 and Ensembl version 92.38. The hallmark and curated gene sets were downloaded from MSigDB (http://www.gsea-msigdb.org/gsea/msigdb/collections.jsp).

The processed data for re-generating all major figures are available through our public GitHub repository at https://github.com/ding-lab/ccRCC_sn_publication/tree/main/plot_data. The remaining data are available within the Article, Supplementary Information, or Source Data file. Source data are provided in this paper.

Human research participants

Policy information about studies involving human research participants and Sex and Gender in Research.

Reporting on sex and gender	We were only able to obtain gender information of the participants, which is based upon self-report, as sex information was not collected. Gender was not considered in the study design. We believe the findings in this study can be applied to more than just one gender, because the participants include both male and female genders (for the snRNA-seq sample set: 18 males and 7 females; for the spatial transcriptomics sample set: one male and one female). The detailed gender information for our main study cohort has been provided in the Supplementary Table 1. We have added a statement in the Methods section as well as Supplementary Table 1 that gender information was collected by CPTAC and the consent was obtained from the participants for sharing individual-level data. We did not perform gender-based analysis as it is not the focus of this study.
Population characteristics	For the snRNA-seq and snATAC-seq samples, patients were diagnosed with clear cell renal cell carcinoma. we have 18 male and 7 female participants based on self-reported gender information. The age distribution is 30-49 (16%), 50-69 (68%), and 70-74 (16%). More demographics, histopathologic information, and treatment details are provided in Supplementary Table 1. For the two spatial transcriptomics samples, sample HT-282N1 was derived from a 65-year-old male with no prior renal cell carcinoma diagnosis and no prior treatment. sample HT-293N1 was derived from a 64-year-old female with prior renal cell carcinoma diagnosis.
Recruitment	For the snRNA-seq and snATAC-seq samples, all patients were recruited through Clinical Proteomic Tumor Analysis Consortium (CPTAC) ccRCC discovery study, in adherence to CPTAC guidelines. There was no potential self selection bias. For the spatial transcriptomics samples, the patients were recruited through the Washington University School of Medicine in St Louis (St Louis, MO). There was no potential self selection bias.
Ethics oversight	Institutional Review Boards (IRBs) of Spectrum Health Services, University of Pittsburgh IRB, Beaumont Health Biobank, International Institute for Molecular Oncology, BioPartners and Asterand Bioscience reviewed protocols and consent documentation, in adherence to the CPTAC guidelines. Informed written consent was obtained from all participants for sharing individual-level data. For the spatial transcriptomics samples, the samples were collected with informed written consent in concordance with the

Washington University Institutional Review Board (IRB) at the Washington University School of Medicine in St Louis (St Louis, MO).

Note that full information on the approval of the study protocol must also be provided in the manuscript.

Field-specific reporting

Please select the one below that is the best fit for your research. If you are not sure, read the appropriate sections before making your selection.

☒ Life sciences ☐ Behavioural & social sciences ☐ Ecological, evolutionary & environmental sciences

For a reference copy of the document with all sections, see nature.com/documents/nr-reporting-summary-flat.pdf

Life sciences study design

All studies must disclose on these points even when the disclosure is negative.

Sample size	We performed snRNA-seq on 34 samples (25 patients) and matching snATAC-seq on 28 of these samples (24 patients) from the Clinical Proteomic Tumor Analysis Consortium (CPTAC) ccRCC collection.<br>Sample size was determined by the availability of remaining sample from the CPTAC ccRCC discovery study (over 100 mg). Another consideration for selecting the sample size is based on the expected number of samples with BAP1 mutation as we intend to study the chromatin accessibility changes associated with BAP1 mutation. BAP1 has been reported to be mutated in 11% of the ccRCC based on the analysis of the TCGA dataset (PMID: 29617669). Thus, we estimated that a cohort of 25 ccRCC patients would contain 2 - 3 BAP1-mutated patients, which would allow us to identify consistent changes associated with BAP1 mutation.
Data exclusions	For the analysis of BAP1-associated differentially accessible chromatin region, one sample with low BAP1 mutation variant allele frequency (<8%) was excluded from the comparison between the BAP1-mutated tumors vs. non-BAP1/PBRM1-mutated tumors.
Replication	For Fig. 1e-f, three independent experiments were performed with similar results.<br>For Fig. 2k, four independent experiments were repeated that shows similar results (as shown in i).<br>For Fig. 5c, two independent experiments were performed with similar results.<br>For Fig. 7e, three independent experiments were performed that showed similar results.
Randomization	The study design was observational only and did not involve allocating patients into treatment groups. Therefore, randomization was not relevant to the study design.
Blinding	The study design was observational only and did not involve allocating patients into treatment groups. Therefore, blinding was not relevant to the study design.

Reporting for specific materials, systems and methods

We require information from authors about some types of materials, experimental systems and methods used in many studies. Here, indicate whether each material, system or method listed is relevant to your study. If you are not sure if a list item applies to your research, read the appropriate section before selecting a response.

Materials & experimental systems

n/a	Involved in the study	
☐	☒	Antibodies
☐	☒	Eukaryotic cell lines
☒	☐	Palaeontology and archaeology
☐	☒	Animals and other organisms
☒	☐	Clinical data
☒	☐	Dual use research of concern

Methods

n/a	Involved in the study	
☒	☐	ChIP-seq
☒	☐	Flow cytometry
☒	☐	MRI-based neuroimaging

Antibodies

Antibodies used	Primary antibodies for IHC:<br>- CA9 Rabbit (#NB100-417; Polyclonal; Novus Bio) at 1:350<br>- CA9 Goat (#PA5-47268, Polyclonal; Invitrogen) at 1:50<br>- VIM Chicken (#NB300-223; Polyclonal; Novus) at 1:150<br>- WNT5a/b Rabbit (#55184-1-AP, Polyclonal; Proteintech) at 1:100<br>- CP Goat (#A80-124A; Polyclonal; Bethyl lab) at 1:100<br>- PCSK6 Rabbit (#PA5-32966; Polyclonal; Invitrogen) at 1:100<br><br>Primary antibodies for WB:

- KLF9 (#sc-376422; Monoclonal (A-5); Santa Cruz) at 1:250
- CP (#A80-124A; Polyclonal; Bethyl lab) at 1:1000
- MXI1 (#12360-1-AP; Polyclonal; Proteintech) at 1:50
- beta-Tubulin (9F3 #2128S; Monoclonal; Cell Signaling Technology) at 1:1000
- BAP1 (#sc-28383; Monoclonal (C-4); Santa Cruz) at 1:500
- beta-Actin (#3700S; Monoclonal (8H10D10); Cell Signaling Technology) at 1:5000

Secondary antibodies
- Licor IR680 Donkey anti-Goat #925-32214, Licor IR680 Donkey anti-Mouse #926-68072 and IR800 Donkey anti-Rabbit #926-32213 fluorescent antibodies were diluted at 1:10,000.
- HRP-conjugated secondary antibody Donkey anti-Mouse #715-035-150 was diluted at 1:10,000.
- Alexa Fluor 488 Donkey anti-Rabbit #711-546-152, Alexa Fluor 488 Donkey anti-Chicken #703-606-155, Alexa Fluor 488 Donkey anti-Goat #705-546-147, Alexa Fluor 594 Donkey anti-Rabbit #711-586-152, Alexa Fluor 594 Donkey anti-Goat #705-586-147, Alexa Fluor 647 Donkey anti-Goat #705-607-003 from Jackson ImmunoResearch were diluted at 1:1000.

Validation

Antibodies are first validated by manufactures where possible. Titration experiment was conducted to determine the optimum concentration of the antibody, where a maximum signal that does not overspill to the adjacent channels could be reached. The validation of these antibodies is completed during the titration experiments. The optimum dilutions of these antibodies are mentioned in the methods section of the manuscript. CA9 (#NB100-417; Novus Bio) rabbit polyclonal antibody validated by manufacturer via IF/IHC/WB and evidences from the citing articles were also listed. e.g. on tissues from human RCC and colorectal cancer (IF).
CA9 (#PA5-47268, Invitrogen) goat polyclonal antibody validated by manufacturer via IF/IHC/WB on human A431 epithelial carcinoma cell line (IF), human colon cancer tissue(IHC) and on human U-87MG glioma cell line and on Hela cell line (WB). In Hela cell line hypoxia was induced by deferoxamine and cobalt chloride. VIM (#NB300-223, Novus) Chicken polyclonal antibody validated by manufacturer via IF/IHC/WB and evidences from the citing articles were also listed. e.g. on tissues from esophageal cancer (IF) and on protein lysate from Hela, SH-SY5Y and NIH-3T3(WB)
Similarly all other antibodies listed above were also validated by the manufacturer in similar manner and thus by articles citing these antibodies.

Eukaryotic cell lines

Policy information about cell lines and Sex and Gender in Research

Cell line source(s)

RCC4 line was purchased from a certified commercial vendor Sigma (https://www.sigmaaldrich.com/US/en/product/sigma/cb_03112702) .
Caki-1 line (catalog number HTB-46, https://www.atcc.org/products/htb-46) and HEK293T (catalog number CRL-3216, https://www.atcc.org/products/crl-3216) were purchased from a certified commercial vendor ATCC.
The SKRC-42 cells were from co-author Dr. James Hsieh's lab https://www.cellosaurus.org/CVCL_6192.

Authentication

RCC4 line was authenticated by STR profiling by Sigma.
Caki-1 line (catalog number HTB-46, https://www.atcc.org/products/htb-46) and HEK293T (catalog number CRL-3216, https://www.atcc.org/products/crl-3216) were authenticated by STR profiling by ATCC.
The SKRC-42 cells were from co-author Dr. James Hsieh's lab https://www.cellosaurus.org/CVCL_6192 and were authenticated by sequencing analysis.

Mycoplasma contamination

All of the cell lines used here were tested negative for mycoplasma contamination.

Commonly misidentified lines
(See ICLAC register)

None of the cell lines used here was among the commonly misidentified lines.

Animals and other research organisms

Policy information about studies involving animals; ARRIVE guidelines recommended for reporting animal research, and Sex and Gender in Research

Laboratory animals

For generating the patient-derived xenograft model, 6-8 week-old female immunodeficient NSG mice (Strain: NOD.Cg-Prkdcscid Il2rgtm1Wjl/SzJ, Stock No: 005557) were purchased from the Jackson Laboratory.

Wild animals

No wild animals.

Reporting on sex

Sex of the animal was not considered in study design as the study only focus on the characteristics of the human tumor, which was implanted on the immunodeficient NSG mice. Furthermore, only female mice was used. Thus, sex-based analyses for the mice was not performed. We believe the findings of this study applies to more than one sex in human, the sex of the mice was irrelevant to our results.

Field-collected samples

No field-collected samples.

Ethics oversight

All animal procedures were reviewed by and received ethical approval from the Institutional Animal Care and Use Committee at Washington University in St. Louis (WUSTL).

Note that full information on the approval of the study protocol must also be provided in the manuscript.
